# A Photoactivable Natural Product with Broad Antiviral Activity against Enveloped Viruses, Including Highly Pathogenic Coronaviruses

**DOI:** 10.1128/AAC.01581-21

**Published:** 2022-02-15

**Authors:** Thomas Meunier, Lowiese Desmarets, Simon Bordage, Moussa Bamba, Kévin Hervouet, Yves Rouillé, Nathan François, Marion Decossas, Valentin Sencio, François Trottein, Fézan Honora Tra Bi, Olivier Lambert, Jean Dubuisson, Sandrine Belouzard, Sevser Sahpaz, Karin Séron

**Affiliations:** a University of Lille, CNRS, INSERM, CHU Lille, Institut Pasteur de Lille, U1019-UMR 9017-CIIL-Center for Infection and Immunity of Lille, Lille, France; b University of Lille, Université de Liège, Université de Picardie Jules Verne, JUNIA, UMRT 1158 BioEcoAgro, Métabolites Spécialisés d’Origine Végétale, Lille, France; c UFR Sciences de la Nature, Université Nangui Abrogoua, Abidjan, Côte d'Ivoire; d University of Bordeaux, CBMN UMR 5248, Pessac, France

**Keywords:** antiviral agents, coronavirus, natural product, virus entry

## Abstract

The severe acute respiratory syndrome coronavirus 2 (SARS-CoV-2) outbreak has highlighted the need for broad-spectrum antivirals against coronaviruses (CoVs). Here, pheophorbide a (Pba) was identified as a highly active antiviral molecule against human CoV-229E after bioguided fractionation of plant extracts. The antiviral activity of Pba was subsequently shown for SARS-CoV-2 and Middle East respiratory syndrome coronavirus (MERS-CoV), and its mechanism of action was further assessed, showing that Pba is an inhibitor of coronavirus entry by directly targeting the viral particle. Interestingly, the antiviral activity of Pba depends on light exposure, and Pba was shown to inhibit virus-cell fusion by stiffening the viral membrane, as demonstrated by cryoelectron microscopy. Moreover, Pba was shown to be broadly active against several other enveloped viruses and reduced SARS-CoV-2 and MERS-CoV replication in primary human bronchial epithelial cells. Pba is the first described natural antiviral against SARS-CoV-2 with direct photosensitive virucidal activity that holds potential for COVID-19 therapy or disinfection of SARS-CoV-2-contaminated surfaces.

## INTRODUCTION

The COVID-19 pandemic has highlighted the lack of specific antiviral compounds available against coronaviruses (CoVs). COVID-19 is caused by the severe acute respiratory syndrome coronavirus 2 (SARS-CoV-2), the third identified human CoV causing severe pneumonia (1–3). Before 2003, coronaviruses were known to cause severe diseases in animals, but human CoVs (HCoVs), such as HCoV-229E and OC-43, were mainly associated with common colds and only rarely with severe outcomes (4). The severe acute respiratory syndrome coronavirus (SARS-CoV) outbreak in 2003 was the first emergence of a highly pathogenic human CoV. The second highly pathogenic coronavirus, identified in 2012 in Saudi Arabia, is the Middle-East respiratory syndrome coronavirus (MERS-CoV), which is still present in the region. SARS-CoV-2 is highly related to SARS-CoV (5). The tremendous efforts of the scientific community worldwide to counteract the COVID-19 pandemic have rapidly led to the development of highly efficient vaccines that are now administered worldwide. Unfortunately, the emergence of SARS-CoV-2 variants in different regions of the world might render the vaccines less efficient and may necessitate a booster vaccination every year, which might not be achievable for billions of humans in all parts of the world. Therefore, to get rid of this pandemic and face future epidemics, it is assumed that not only vaccination but also efficient antiviral treatments are necessary. Before the emergence of SARS-CoV-2, no specific antiviral was commercially available for treatment of CoV infections. Due to the urgent need for antivirals against SARS-CoV-2, many researchers have focused their investigations on repurposing available drugs. Unfortunately, until now, none of them has been able to significantly reduce severe outcomes in patients. High-content screening *in vitro* of approved drugs identified some interesting antiviral molecules that still have to be tested in clinics on patients with COVID-19 (6–8). Protease and polymerase inhibitors are also widely investigated in *in vitro* studies (9). Recently, White et al. identified plitidepsin, an inhibitor of the host protein eEF1A, as a potential antiviral agent for SARS-CoV-2 (10). To date, only synthetic neutralizing monoclonal antibodies have been approved for emergency use in newly infected patients.

Coronaviruses are members of the *Coronaviridae* family within the order *Nidovirales.* CoVs are enveloped viruses with a positive single-stranded RNA genome of around 30 kb. The genome encodes 4 structural proteins, the nucleocapsid (N), the spike (S), the envelope (E), and the membrane (M) proteins. The S protein is important for the interaction of the viral particle with the cellular host receptor, being angiotensin-converting enzyme 2 (ACE2) for SARS-CoVs (11, 12), dipeptidyl peptidase 4 (DPP4) for MERS-CoV (13), and aminopeptidase N (APN) for HCoV-229E (14). Once attached, the virus releases its genome into the cytosol by fusion of the viral envelope with a host membrane. This fusion process is mediated by the S protein, a class I fusion protein, and can occur either at the plasma or at the endosomal membrane. Viral class I fusion proteins are typically synthesized as inactive precursor proteins and require proteolytic activation by cellular proteases to acquire their fusion-competent state. The host cell protease TMPRSS2 has been shown to be necessary for plasma membrane fusion of many coronaviruses, including SARS-CoV-2 (12, 15, 16), whereas cathepsins are often involved in fusion processes at endosomal membranes (17).

It is estimated that about 80% of the global population relies on traditional medicine to treat infectious diseases. Plants are a natural source of compounds with a structural diversity that is much higher than those obtained by chemical synthesis. Many of these compounds have proven their antiviral activity *in vitro*. To date, some reports describe the antiviral activity of natural compounds on coronavirus, including SARS-CoV-2, but many of them are *in silico* analyses without any *in vitro* or *in vivo* evidence (18, 19).

Here, we show that pheophorbide a (Pba), isolated from *Mallotus oppositifolius* (Geiseler) Müll.Arg. (Mo, *Euphorbiaceae*) leaf crude extract after bioguided fractionation, has antiviral activity against various CoVs, including HCoV-229E, MERS-CoV, and SARS-CoV-2, and against other enveloped viruses, such as yellow fever virus (YFV), hepatitis C virus (HCV), and Sindbis virus (SINV). Moreover, we demonstrate that Pba is an antiviral photosensitizer directly acting on the viral particle, thereby impairing the virus-cell fusion step.

## RESULTS

### Pba isolated from crude methanolic extract from *Mallotus oppositifolius* (Geiseler) Müll.Arg. is highly active against HCoV-229E.

**FIG 1:**
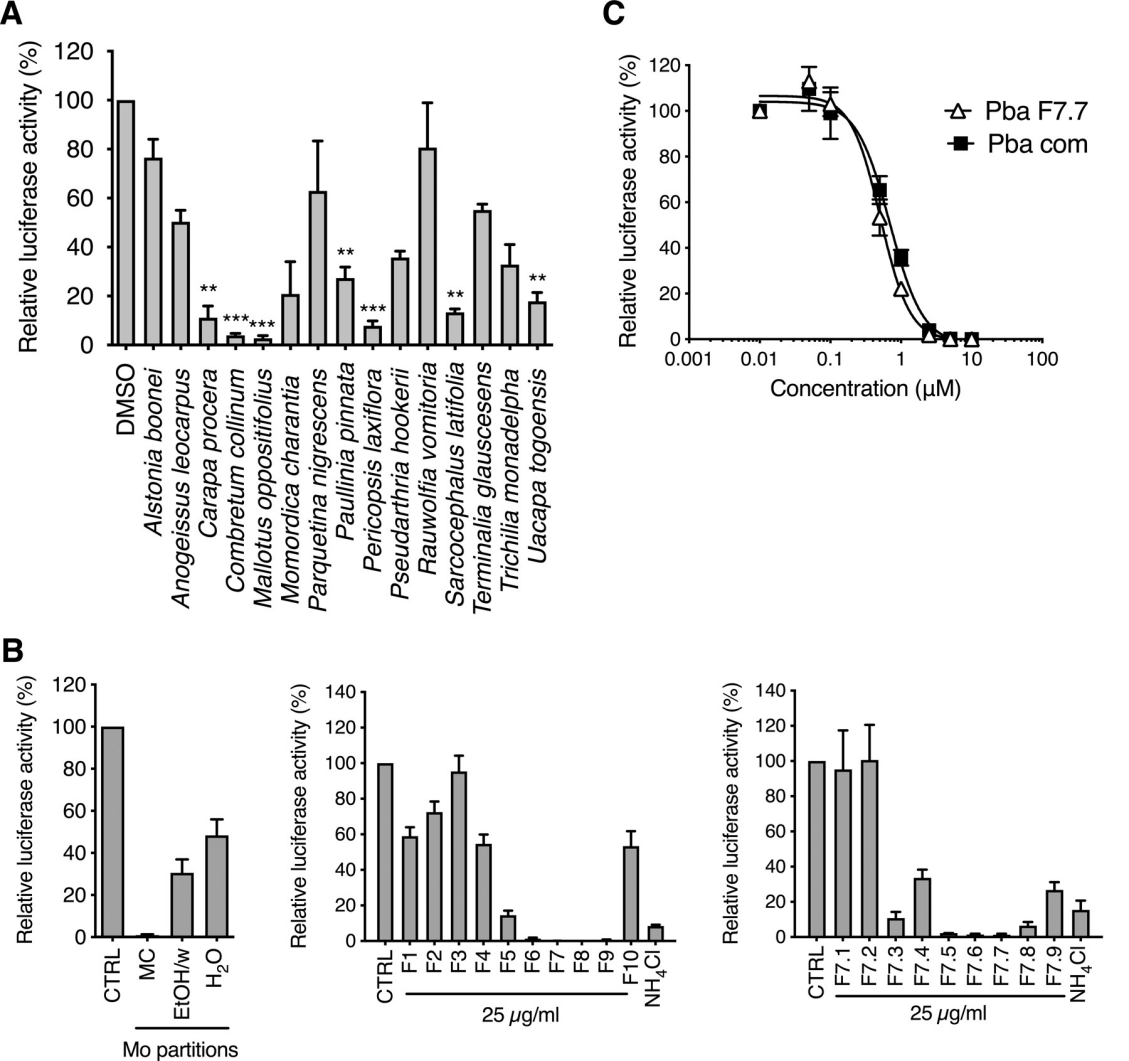
Identification of Pba as active compound in *Mallotus oppositifolius* using bioguided fractionation of plant extracts. (A) Huh-7 cells were inoculated with HCoV-229E in the presence of various plant extracts at 25 μg/ml. Cells were lysed 7 h postinoculation and luciferase activity quantified. (B) Huh-7 cells were inoculated with HCoV-229E in the presence of subextracts of Mo (methylene chloride, MC; ethanol-water [50:50], EtOH/w; water, H_2_O; left), fractions of Mo MC subextract (middle), or subfractions of F7 fraction (right) at 25 μg/ml. Cells were lysed 7 h postinoculation and luciferase activity quantified. (C) Huh-7 cells were inoculated with HCoV-229E-Luc in the presence of Pba extracted from Mo (Pba F7.7) or commercial Pba (Pba com) at different concentrations. At 1 h p.i., cells were washed and fresh compounds were added to the cells for 6 h, after which cells were lysed to quantify luciferase activity. Data are expressed relative to the control DMSO. Results are expressed as mean ± standard errors of the means (SEM) from 3 experiments.

Fifteen plant methanolic extracts from the Ivorian pharmacopeia, which were initially screened for their anti-HCV activity (M. Bamba, S. Bordage, M.-E. Sahuc, S. Moureu, J. Samaillie, V. Roumy, P. Vauchel, K. Dimitrov, Y. Rouillé, J. Dubuisson, F. H. Tra Bi, K. Séron, and S. Sahpaz, unpublished data), were tested against HCoV-229E-Luc, a luciferase recombinant version of HCoV-229E, which allows for rapid and easy screening of diverse molecules *in vitro*. Seven of the 15 extracts significantly reduced HCoV-229E infection (Fig. 1A), whereas none of the extracts showed cytotoxicity in Huh-7 cells at the tested concentrations (Bamba et al., unpublished). The *Mallotus oppositifolius* (Mo) crude extract was the most active and therefore was selected for further analyses. A bioguided fractionation was performed to determine the active compound(s) in this plant. This revealed that the methylene chloride (MC) partition was the most active on HCoV-229E (Fig. 1B) and therefore was chosen for fractionation by centrifugal partition chromatography (CPC), leading to 10 fractions (F1 to F10). Among these fractions, F7 was the most active and subjected to further fractionation by preparative high-performance liquid chromatography (HPLC), leading to 9 subfractions (F7.1 to F7.9). F7.7 was the most active of them and seemed to contain only one molecule. This dark green product was analyzed by LC-ultraviolet-mass spectrometry (LC-UV-MS), which revealed only one peak (m+H = 593.3) with two maxima of absorption in visible light (409 and 663 nm). This information was used for a Dictionary of Natural Products search (https://dnp.chemnetbase.com/), indicating that this molecule could be pheophorbide a (Pba). F7.7 purity and chemical structure were further confirmed by nuclear magnetic resonance (NMR) (data not shown).

To confirm that Pba was the active compound of the Mo extract, a dose-response experiment was performed with both the pure compound isolated in F7.7 and commercial Pba against HCoV-229E-Luc in Huh-7 cells. As shown in Fig. 1C, 50% inhibitory concentration (IC_50_) values were comparable for both natural and commercial Pba (0.51 μM and 0.54 μM, respectively), confirming that Pba was indeed the active substance of Mo.

### Pba is active against several human CoVs at noncytotoxic concentrations.

**FIG 2:**
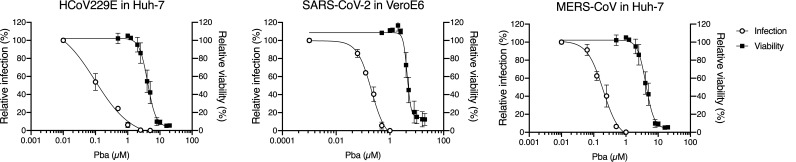
Pba inhibits various HCoVs. For infection assays, cells were inoculated with HCoV-229E (Huh-7 cells), SARS-CoV-2 (Vero-E6 cells), and MERS-CoV (Huh-7 cells) in the presence of various concentrations of Pba. At 1 h p.i., cells were washed and fresh compounds were added to the cells for 9 h (HCoV-229E) or 16 h (SARS-CoV-2 and MERS-CoV) and the supernatants were collected for infectivity titration. For toxicity assays, cells were incubated with Pba at different concentrations for 24 h. MTS assay was performed to monitor cell viability. For both infection and toxicity tests, cells were exposed to the light of the cabinet for 10 min, once upon inoculation, and once after 1 h of incubation. Results are expressed as means ± SEM from 3 experiments.

Prior to the assessment of its broad-spectrum activity against various CoVs, the cytotoxicity of Pba was determined in different cell lines. As Pba is known to be a photosensitizer, it was assumed that light could affect its toxicity. MTS [3-(4,5-dimethylthiazol-2-yl)-5-(3-carboxymethoxyphenyl)-2-(4-sulfophenyl)-2H-tetrazolium] assays were performed under conditions similar to those for the infection procedures. Cells were incubated with Pba at different concentrations and taken out of the incubator after 1 h to change the medium and left for 10 min under light exposure in a biosafety cabinet (BSC), after which they were replaced in the incubator for 23 h. In parallel, plates were kept for 24 h in the dark. Pba did not exhibit any toxicity at concentrations up to 120 μM when left in the incubator for 24 h without light exposure, whereas it showed some toxicity when the cells were shortly exposed to light, with 50% cytotoxic concentration (CC_50_) values of 4.4 ± 1.2 μM, 5.8 ± 1.9 μM, and 5.5 ± 2.0 μM for Huh-7, Vero-E6, and Vero-81 cells, respectively (Fig. 2; see also Fig. S1 in the supplemental material). Taken together, these results show that the cytotoxicity of Pba in cell culture depends on light exposure.

The antiviral activity of Pba was tested on different human CoVs. It was first confirmed on HCoV-229E by measuring infectious titers (Fig. 2) with an IC_50_ value of 0.1 μM, resulting in a selectivity index (SI) of 44. Similarly, infection inhibition assays were performed with various noncytotoxic concentrations of Pba against SARS-CoV-2 and MERS-CoV. As shown in Fig. 2, Pba had a strong antiviral effect against both highly pathogenic coronaviruses at nontoxic concentrations, with IC_50_ values of 0.18 μM for both SARS-CoV-2 and MERS-CoV, resulting in a selectivity index of 32 and 24, respectively.

### Pba is an inhibitor of coronavirus entry by direct action on the particle.

**FIG 3:**
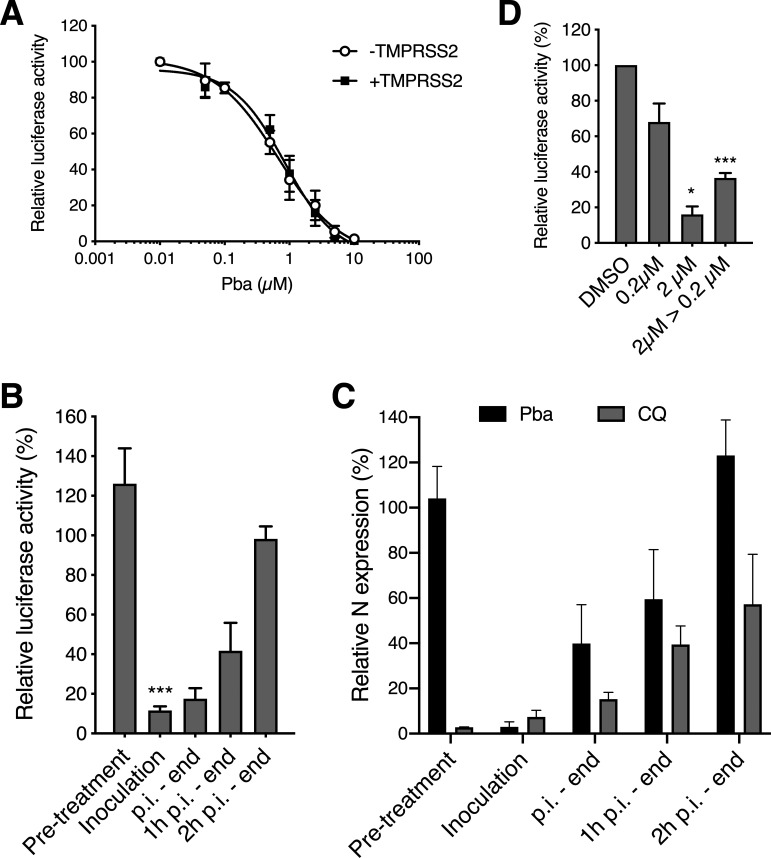
Pba inhibits viral entry by a direct action on the viral particle. (A) Huh-7 and Huh-7-TMPRSS2 cells were inoculated with HCoV-229E-Luc in the presence of various concentrations of Pba. At 1 h p.i., cells were washed and fresh compounds were added to the cells for 6 h, after which the cells were lysed to quantify luciferase activity. Data are expressed relative to the control, DMSO. (B) Pba at 2 μM was added at different time points during infection of Huh-7-TMPRSS2 cells by HCoV-229E-Luc, either 1 h before inoculation (pretreatment), for 1 h during inoculation (inoculation), for 6 h postinoculation (p.i.-end), after 1 h postinoculation until the end (1 h p.i.-end), or 2 h postinoculation until the end (2 h p.i.-end). Cells were lysed 7 h after the inoculation and luciferase activity quantified. (C) A similar experiment was performed in Vero-81 cells inoculated with SARS-CoV-2 in the presence of Pba at 1 μM or chloroquine (CQ) at 10 μM at different time points. Cells were lysed 16 h postinoculation, and the viral nucleocapsid protein was detected by Western blotting. The graph represents the quantification of the band intensity corresponding to the N protein relative to the DMSO control for each time point. (D) Huh-7 cells were inoculated with HCoV-229E-Luc in the presence of 0.2 or 2 μM Pba or with HCoV-229E-Luc previously treated with 2 μM Pba and then diluted 10 times, leading to a concentration of 0.2 μM Pba for the inoculation period (2 μM to 0.2 μM). The amount of virus used for inoculation was kept constant for the different conditions, and all the samples were exposed to the light for 10 min. At 7 h postinoculation, cells were lysed and luciferase activity was quantified. *, *P* < 0.05; ***, *P* < 0.005.

Coronaviruses fusion is triggered by proteolytic cleavage of the spike protein. Depending on cellular proteases available, fusion can occur after endocytosis of the virus or directly at the cell surface. It has been demonstrated that HCoV-229E and SARS-CoV-2 fusion at the plasma membrane depends on the expression of the TMPRSS2 protease (12, 20), and for many coronaviruses entry via fusion at the plasma membrane has been shown to be the most relevant pathway *in vivo* (21). To determine if Pba was able to inhibit both entry pathways, its antiviral activity was tested in Huh-7 and Huh-7-TMPRSS2 cells, the latter being obtained after transduction with a TMPRSS2 lentiviral expression vector. Two inhibitors of the entry pathway were used as a control, E64D as inhibitor of the endocytic pathway and Camostat as inhibitor of the TMPRSS2 protease. As shown in Fig. S2, E64D specifically inhibited HCoV-229E infection in Huh-7 cells, whereas Camostat inhibited infection in Huh-7-TMPRSS2 cells. Complete inhibition of infection was observed when a combination of the two inhibitors was used, showing that both entry pathways can be used *in vitro*. No difference in antiviral activity of Pba against HCoV-229E infection was observed in the presence or absence of TMPRSS2 (Fig. 3A). Similar results were obtained with SARS-CoV-2 in Vero cells (our unpublished observation). These results show that Pba exhibits antiviral activity whatever the entry pathway used.

To gain insights into the mechanism of action of Pba, a time-of-addition assay with Pba was performed during HCoV-229E or SARS-CoV-2 infection. Therefore, Pba was added at different time points before, during, or after inoculation. All experiments were performed under the BSC’s light exposure. For both viruses, no inhibition of infection was observed when Pba was added to the cells before inoculation (pretreatment of the cells), whereas a strong inhibition of infection was noticed when Pba was present during the virus inoculation step (Fig. 3B and C). When Pba was added only after inoculation, its inhibitory effect rapidly dropped and normal levels of infectivity were observed again when Pba was added more than 2 h after inoculation. Chloroquine is a well-known inhibitor of SARS-CoV-2 replication *in vitro*, as it inhibits virus-cell fusion after endocytosis by preventing endosomal acidification. As shown in Fig. 3C, the Pba and chloroquine inhibition curves were rather similar, showing that Pba could inhibit virus entry. It is worth noting that normal levels of infectivity were observed when Pba was added more than 2 h after inoculation, whereas chloroquine-treated cells remained at around 60% of normal levels, which may be due to inhibition of the second round of entry, since our experimental conditions are compatible with reinfection. Taken together, these results suggest that Pba is inhibiting virus entry.

Since no antiviral activity was observed with cells treated with Pba prior to inoculation, we wondered whether Pba targets the virus instead of the cells. To test this hypothesis, HCoV-229E-Luc was preincubated for 30 min at a concentration 10 times higher than that used during inoculation. For this experiment, HCoV-229E-Luc was preincubated with Pba at 2 μM for 30 min and then diluted to reach a concentration of Pba of 0.2 μM for inoculation, a concentration that does not severely impact HCoV-229E-Luc infection, as shown above. In parallel, cells were directly inoculated with HCoV-229E-Luc at 0.2 and 2 μM as a control. The results clearly show that when HCoV-229E-Luc was pretreated with Pba at high concentration (2 μM) before inoculation at low concentration (0.2 μM), the antiviral activity was much stronger than that when inoculation was performed in the presence of 0.2 μM Pba without any pretreatment (Fig. 3D). Taken together, these results indicate that Pba inhibits HCoV entry by a direct effect on the viral particle.

### Pba is an inhibitor of viral fusion.

**FIG 4:**
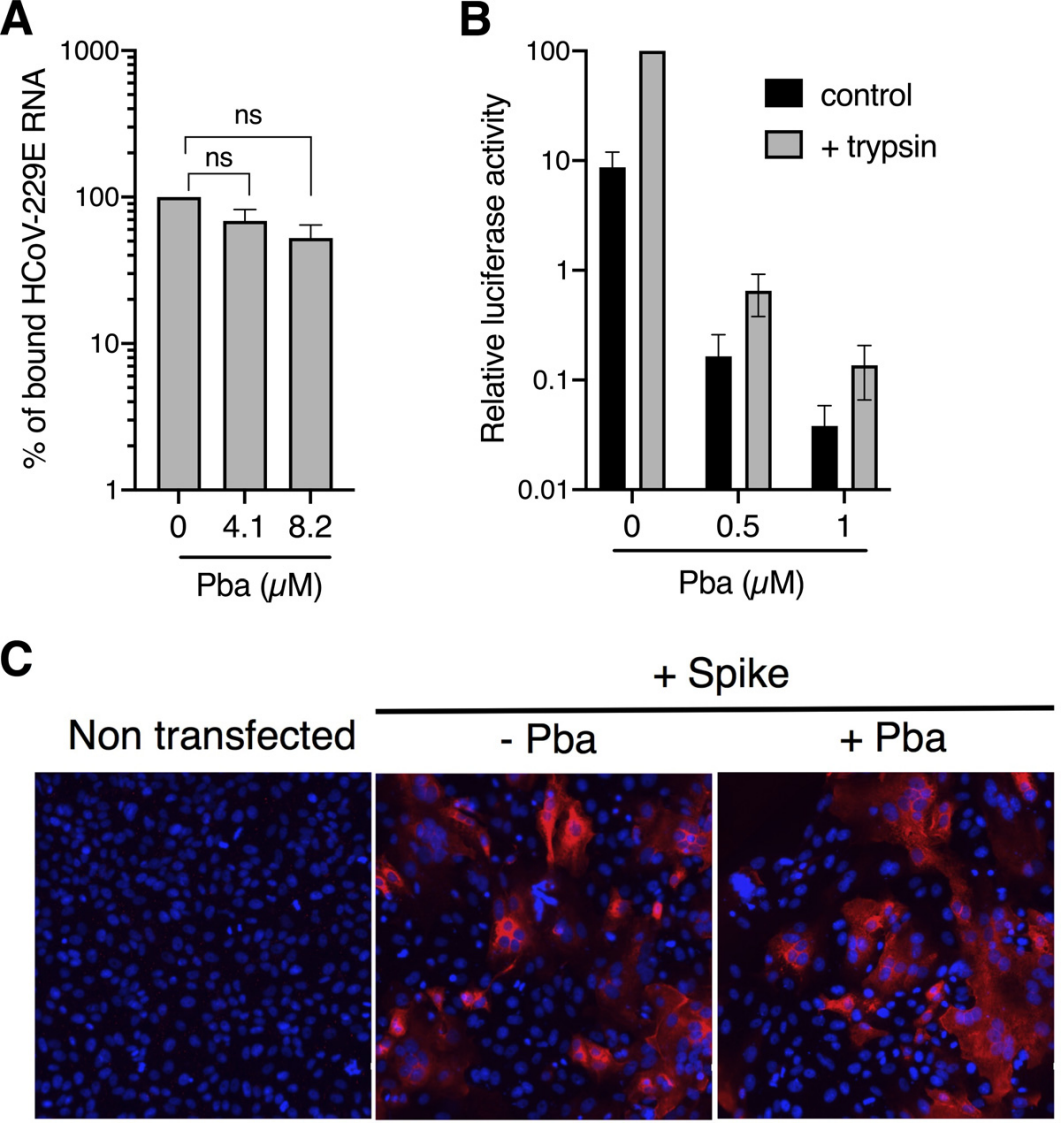
Pba inhibits viral entry at the fusion step. (A) Huh-7-TMPRSS2 cells were inoculated with HCoV-229E for 1 h at 4°C in the presence of DMSO or 4.1 and 8.2 μM Pba. Cells were washed thrice with ice-cold PBS, and total RNA was extracted. Bound HCoV-229E virions were detected by quantification of HCoV-229E gRNA by qRT-PCR. Relative binding is expressed as the percentage of the control (DMSO), for which the 100% value was arbitrarily attributed. Mean values ± SEM (error bars) from three independent experiments are presented. n.s., not significant. (B) HCoV-229E was incubated with Pba at different concentrations and was bound to Huh-7 cells for 1 h in the absence (control) or presence (trypsin) of NH_4_Cl at 4°C. Under the later condition, fusion was induced by 3 μg/ml trypsin for 5 min at 37°C in the presence of NH_4_Cl. Cells were lysed 7 h postinfection and luciferase activity quantified. Infectivity is expressed as the percentage of the control (DMSO) for which the 100% value was arbitrarily attributed. Mean values ± SEM (error bars) from three independent experiments are presented. (C) Vero-81 cells transiently expressing SARS-CoV-2 spike protein were incubated with or without Pba at 1 μM from 6 to 24 h p.i., after which syncytia were visualized by immunofluorescence. Images were acquired on an Evos M5000 imaging system (Thermo Fisher Scientific).

Virus entry can be divided into two different steps, first the viral attachment to the cell surface and second the fusion of the virus envelope with cellular membranes. To further define the mode of action of Pba, experiments were performed with HCoV-229E, which can be manipulated in a lower-level containment facility. To determine a potential effect at the attachment step, Huh-7-TMPRSS2 cells were incubated with HCoV-229E in the presence or absence of Pba at 4°C for 1 h. These conditions block endocytosis but allow virus attachment to the cell surface. Cells were rinsed with phosphate-buffered saline (PBS), and the amount of virions attached to the surface was determined by quantification of viral genomes by quantitative reverse transcription-PCR (qRT-PCR). As shown in Fig. 4A, only a slight and nonsignificant decrease in RNA levels was observed in the presence of Pba, indicating that Pba barely affects virus attachment. The action of Pba on the fusion step was investigated in virus-cell fusion assay by using trypsin as an exogenous protease to induce coronavirus membrane fusion at the plasma membrane (22, 23). Huh-7 cells were treated with NH_4_Cl to inhibit fusion in the endocytic pathway, and viruses were bound at the cell surface at 4°C. The fusion then was induced by a short trypsin treatment at 37°C. Entry at the cell surface was more efficient in the presence of trypsin than the control (Fig. 4B), which is consistent with other reports (22, 23). In addition, Pba at both 0.5 and 1 μM strongly inhibited infection levels under trypsin-mediated fusion conditions in a range similar to that of the inhibition seen with the control. Taken together, these results indicate that Pba inhibits entry at the fusion step and not by preventing attachment.

Two viral structures are involved in virus-cell fusion, namely, the spike protein and the viral membrane itself. To find out if Pba targets the spike, a cell-cell fusion assay was performed. For this, the SARS-CoV-2 spike protein was transiently expressed by plasmid transfection in Vero-81 cells. At 6 h posttransfection (p.t.), the medium was replaced with medium containing either dimethyl sulfoxide (DMSO) or 1 μM Pba until 24 h p.t. As shown in Fig. 4C, spike-induced cell-cell fusion (apparent as syncytium formation) occurred equally well under control (DMSO)- and Pba-treated conditions, indicating that Pba cannot prevent cell-cell fusion induced by the viral spike protein. Taken together, these results are not in favor of an effect of Pba on the viral spike fusion protein.

### The antiviral activity of Pba is increased upon light exposure and targets the viral membrane.

**FIG 5:**
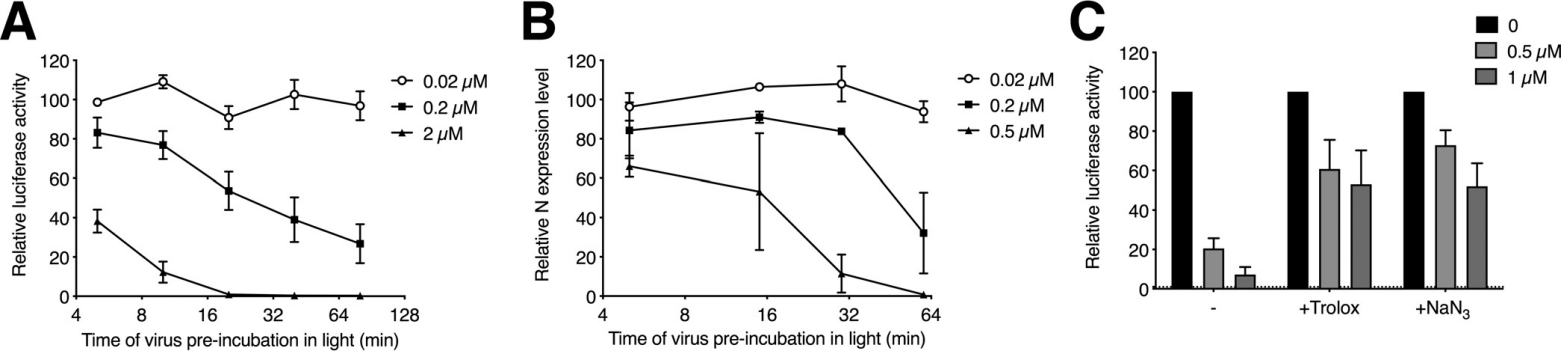
Antiviral activity of Pba depends on light exposure and its ability to generate singlet oxygen species. HCoV-229E-Luc (A) and SARS-CoV-2 (B) were incubated with Pba at given concentrations under the light of the laminar flow cabinet. At different time points of light exposure, the mixture was used to inoculate Huh-7 cells or Vero-81 cells, respectively. At 7 h (HCoV-229E-Luc) or 16 h (SARS-CoV-2) postinoculation, cells were lysed and infection quantified as described previously. (C) Pba at 0.5 or 1 μM was mixed with 10 mM Trolox or 10 mM NaN_3_ in DMEM, and HCoV-229E-Luc was added to the mixture prior to inoculation of Huh-7 cells for 1 h at 37°C. Inoculum and compounds were removed and replaced with culture medium for 6 h. Cells were lysed and luciferase activity quantified. Infectivity is expressed as the percentage of infection relative to the control (DMSO) to which the 100% value was arbitrarily attributed. Mean values ± SEM (error bars) from three independent experiments are presented.

Given that Pba is photoactivable, we wondered whether its antiviral effect is light dependent. We therefore inoculated Huh-7 cells with HCoV-229E in the presence of Pba under different light exposure conditions. As shown in Fig. S3, the antiviral activity of Pba increased upon light exposure. However, even in the dark, antiviral activity was observed at 10 μM. A typical feature of photosensitizers is that the concentration required for its biological properties decreases upon increase of time of exposure to the light. To see whether Pba behaves like other photosensitizers, HCoV-229E was preincubated with Pba at various concentrations (0.02, 0.2, and 2 μM) and exposed to the normal white light of the laminar flow cabinet for different durations (ranging from 5 to 80 min). It is important to note that light alone was not responsible of the antiviral activity, because there was no antiviral effect after a long exposure time (80 min) with Pba at an inactive concentration (0.02 μM). As shown in Fig. 5A, results clearly showed that with the same concentration of Pba, increased inhibitory effect could be observed with longer light exposure times. Similar results were observed with SARS-CoV-2 (Fig. 5B). Together, these data indicate that the anticoronavirus properties of Pba depend on its dynamic photoactivation.

Photodynamic inactivation (PDI) of microorganisms typically results from the onset of reactive oxygen species (ROS), including free radicals or singlet oxygen species (^1^O_2_), generated when the light-activated photosensitizer falls back to its ground state, thereby transferring its energy to molecular oxygen (resulting in the onset of ^1^O_2_) or initiating photochemical reactions with ROS generation. Two mechanisms of activation have been described, either type I reactions in which the photosensitizer activates a substrate that generates ROS or type II reactions in which the photosensitizer directly generates ^1^O_2_. Subsequently, these species can damage various microorganism structures, such as nucleic acids, proteins, or lipids (24, 25). To determine if the antiviral activity of Pba also depends on ROS or ^1^O_2_ generation, infection was performed in the presence of quenchers that are able to trap these generated oxygen species. Two ^1^O_2_ quenchers were used, a water-soluble analogue of vitamin E, Trolox, and NaN_3_ (Fig. 5C). HCoV-229E was mixed with the quenchers Trolox and NaN_3_, both at 10 mM, and Pba was added at the inoculation step. The cells were rinsed and fresh culture medium was added for 6 h. The results clearly showed that both Trolox and NaN_3_ were able to reduce the action of Pba (Fig. 5C), indicating that the antiviral activity of Pba is mediated by the generation of ^1^O_2_ after photoactivation.

**FIG 6:**
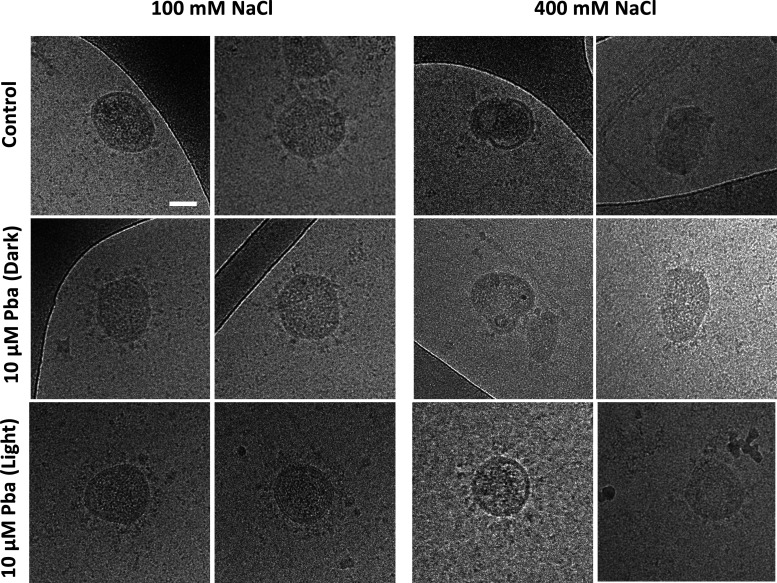
Pba renders virions resistant to osmotic shock. HCoV-229E were incubated in the presence or absence of Pba at 10 μM with or without 30 min light exposure, after which the particles were subjected to normal medium conditions (100 mM NaCl) or to an osmotic shock of 400 mM NaCl for 30 s. Virions were fixed with PFA and samples were treated for cryo-EM observation. Images are representative of 30 independent images of 2 independent experiments. Scale bar, 50 nm.

Vigant et al. clearly demonstrated that the generation of ^1^O_2_ by the lipophilic photosensitizer LJ001 induces the phosphorylation of unsaturated phospholipid of viral membranes and changes the biophysical properties of viral membranes, thereby affecting membrane fluidity and/or increasing rigidity (24). As a result, the change of fluidity and/or rigidity of the viral membrane impairs its ability to undergo virus-cell fusion. We therefore wondered whether a similar action of the photosensitizer on the lipids of the viral envelope might also explain our observation that Pba is able to inhibit HCoV-229E fusion by targeting the viral particle. We hypothesized that the Pba-induced membrane rigidity renders the virus less sensitive to a shrinkage effect induced by an osmotic stress. Therefore, HCoV-229E was incubated with Pba either in the dark or under light exposure for 30 min and subjected to osmotic shock with 400 mM NaCl before fixation with 4% paraformaldehyde (PFA). Fixed viral particles were visualized by cryoelectron microscopy (cryo-EM). As shown in Fig. 6, intact virions with their characteristic spikes at the surface can be observed under untreated conditions (control). The addition of Pba either in the dark or under light conditions did not affect the overall morphology of virions under normal medium conditions. Interestingly, when intact virions were subjected to an osmotic stress by increasing NaCl concentration from 100 mM to a final concentration of 400 mM, the virions shape was altered due to shrinkage of the viral membrane. When virions incubated with Pba in the dark were subjected to osmotic shock, a similar alteration of viral shape was observed. However, in the presence of Pba and under light conditions, no membrane deformation was observed, suggesting that the virus was more resistant to osmotic shock. These results clearly show that light-activated Pba modifies the mechanical properties of the viral envelope by increasing its stiffness. This Pba-induced increase in envelope rigidity likely prevents the membrane deformation needed to undergo virus-cell fusion. Knowing that there might be an effect of the light, the spike-induced cell-cell fusion assay as shown in Fig. 4C was repeated with short light exposure of the spike-transfected cells every 2 h after addition of Pba. Even with regular exposure to light, no effect of Pba was seen on the spike-mediated cell-cell fusion (data not shown), further excluding that Pba additionally targets the spike protein.

### Pba is a broad-spectrum antiviral that targets viral membranes of several enveloped viruses.

**FIG 7:**
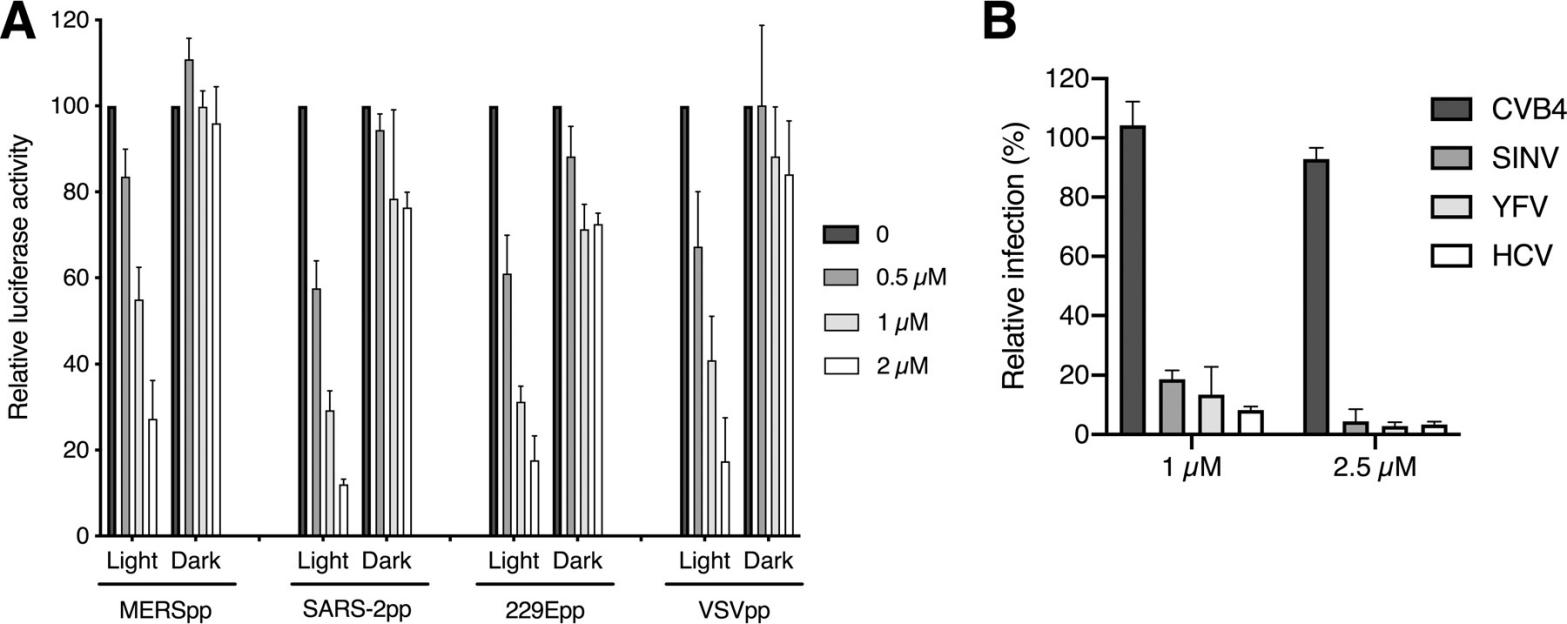
Pba is a broad-spectrum antiviral agent against enveloped viruses. (A) MERSpp, HCoV-229Epp, VSVpp, and SARS-2pp were preincubated with Pba at the indicated concentration either under light for 30 min (light) or without light (dark) prior to inoculation of Huh-7 cells expressing ACE2 and TMPRSS2 for 2 h. At 46 h postinoculation, cells were lysed and luciferase activity was quantified. Infectivity is expressed as the percent relative to the control (DMSO), to which the 100% value was arbitrarily attributed. Mean values ± SEM (error bars) from three different experiments are presented. (B) Different viruses were incubated with Pba at 1 and 2.5 μM under light condition for 30 min prior to inoculation. Cells were fixed at different time points depending on the virus (see Materials and Methods for details) and subjected to immunofluorescence labeling. Infectivity is expressed as percent relative to the control (DMSO). Mean values ± SEM (error bars) from three different experiments are presented.

In contrast to viral proteins, viral membranes are derived from the host cell and, hence, are not virus specific. This suggests that Pba has broader antiviral activity against enveloped viruses. Indeed, antiviral activities against other viruses have already been reported for Pba or related molecules. These include hepatitis C virus (HCV), HIV (human immunodeficiency virus), and IAV (influenza A virus) (26–28). To confirm the broad-spectrum activity of Pba, its antiviral activity was tested on pseudotyped viral particles with envelope proteins of VSV (vesicular stomatitis virus), HCV, SARS-CoV-2, and MERS-CoV. Pseudoparticles were pretreated with 0.5, 1, or 2 μM Pba and left in the dark or exposed to light for 30 min prior to inoculation. The results clearly showed that Pba inhibited pseudoparticle infection regardless of the nature of the viral envelope protein but only under light conditions (Fig. 7A). Next, the antiviral activity of Pba was tested for coxsackievirus (CVB4; nonenveloped), Sindbis virus (SINV; enveloped), hepatitis C virus (enveloped), and yellow fever virus (YFV; enveloped). Whereas Pba was not active on the nonenveloped virus CVB4, it showed a clear antiviral effect against the 3 enveloped viruses HCV, YFV, and SINV (Fig. 7B). Taken together, these results confirm the light-dependent activity of Pba on enveloped viruses and suggest that the lipid membrane is the most likely target of the compound.

### Other chlorophyll-derived products and photosensitizers possess a light-dependent anticoronaviral activity.

Pba is a breakdown product of chlorophyll. Chlorophyll is metabolized into different compounds, including Pba, pyropheophorbide a (pyroPba), and chlorin e6. We wondered if these products would also have antiviral activity. Furthermore, we selected nine porphyrins or metalloporphyrins structurally related to Pba (*N*-methyl protoporphyrin IX, *N*-methyl mesoporphyrin IX, Zn-protoporphyrin IX, tin-mesoporphyrin IX, temoporfin, phthalocyanine, hemin chloride, HPPH, and 5,15-DPP) and one photosensitizer without related structure (Rose Bengale) to determine if similar antiviral activity against coronaviruses could be identified. The toxicity and antiviral activity of these compounds were investigated (Fig. S4). Chlorophyll *b*, phthalocyanine, and 5,15-DPP were not active at the tested concentrations. Three molecules, hemin chloride, temoporfin, and Rose Bengale, had moderate antiviral activity. The antiviral activity of the six most active compounds was tested both under normal white light conditions and in the dark, clearly showing that, similar to Pba, the antiviral activity of most molecules tested was also light dependent (Fig. S3). Interestingly, *N*-methyl protoporphyrin IX and *N*-methyl mesoporphyrin IX showed antiviral activity under dark conditions but much less than that under light exposure. PyroPba was toxic at tested concentrations; thus, dose-response experiments with lower concentrations were performed to determine the precise CC_50_ and IC_50_. This was also done for all the active compounds. The CC_50_ and IC_50_ of the different compounds were compared, and only pyroPba was more active against HCoV-229E than Pba (Table 1), with an IC_50_ of 0.35 μM. However, this compound is also more toxic, with a CC_50_ of 2.67 μM and a selective index of 7.6. Thus, our results show that porphyrin-related compounds have antiviral activity against HCoV-229E but that Pba and pyroPba are the most active under normal white light exposure.

**TABLE 1:** Inhibitory and cytotoxic concentrations, and selective index of photosensitizers against HCoV-229E

Molecule	IC_50_ (μM)	CC_50_ (μM) 24 h	SI
Pba	0.54	6.08	11.2
PyroPba	0.35	2.67	7.6
Chlorin e6	0.72	21.4	29.7
HPPH	1.14	8.13	7.1
*N*-Methyl protoporphyrin IX	1.21	ND > 80	>66.1
*N*-Methyl mesoporphyrin IX	1.25	ND > 80	>64
Zn protoporphyrin IX	0.79	16.64	21.0
Rose Bengale	2.86	ND > 80	27.9

### Pba reduces SARS-CoV-2 and MERS-CoV replication in human primary airway epithelial cells.

**FIG 8:**
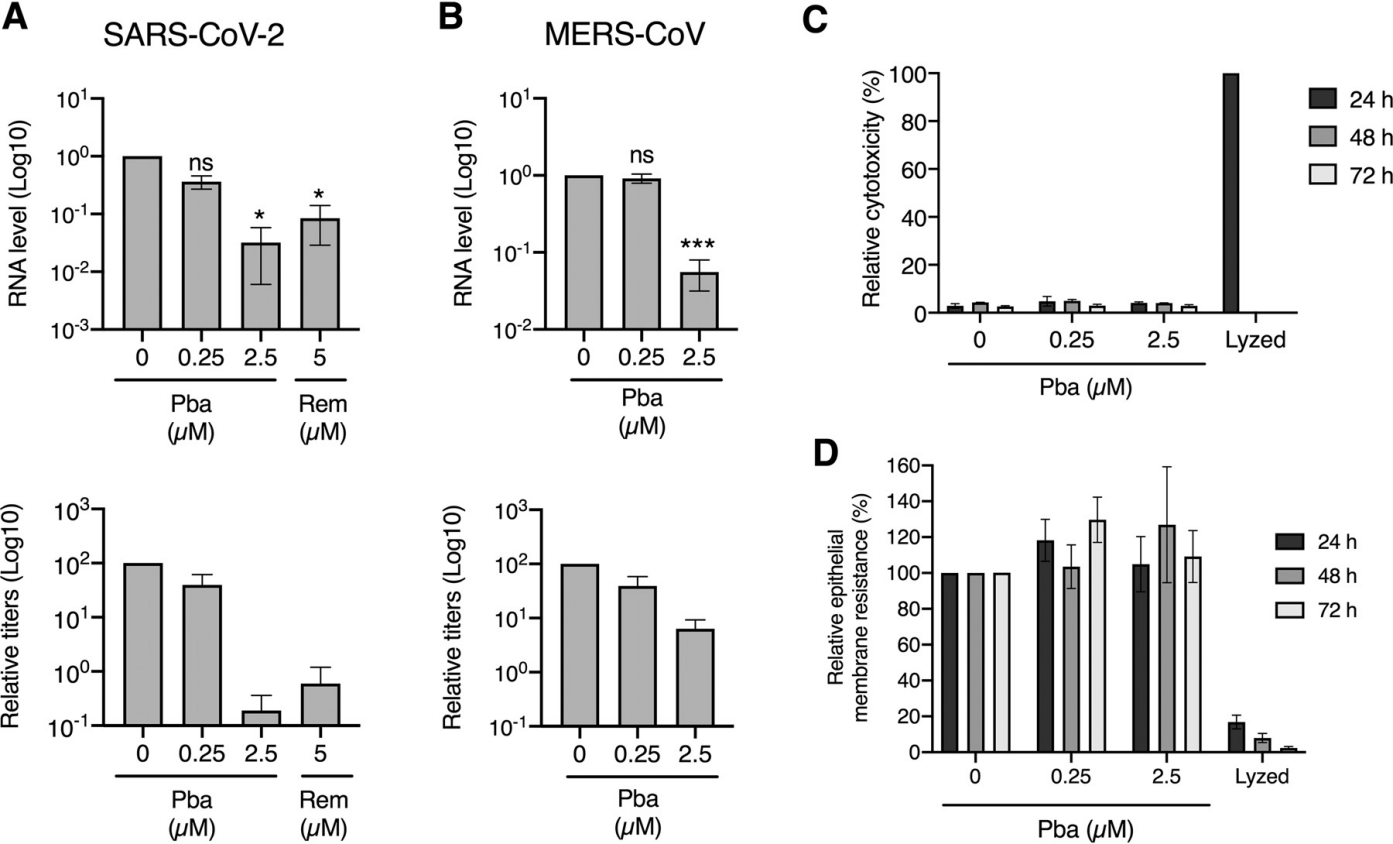
Antiviral efficacy of Pba in human primary bronchial epithelial cells. Mucilair cells were inoculated with SARS-CoV-2 or MERS-CoV in the presence of Pba at 0.25 or 2.5 μM for 1 h at the apical side. The cells were left for 10 min under the light of the cabinet before being put in the incubator. Inoculum was removed 1 h postinoculation. Remdesivir (Rem) at 5 μM was added in the basolateral medium. The cells were additionally exposed to the light for 10 min at 24 h, 48 h, and 72 h postinoculation. At 72 h postinoculation, viruses were collected from the apical surface, and cells were lysed to extract RNA. Viral RNA was quantified by qRT-PCR, and viral titers were determined by infectivity titrations for SARS-CoV-2 (A) and MERS-CoV (B). For RNA quantification, data are expressed relative to the control, DMSO. Results are expressed as mean ± SEM from 3 experiments. Viral titers are representative of three independent experiments. Toxicity of Pba was measures in Mucilair treated with Pba at 0.25 and 2.5 μM by measuring either LDH secretion (C) or transepithelial electrical resistance (D) at 24 h, 48 h, and 72 h of incubation. As a control, cells were lysed with lysis solution as described in Materials and Methods. Results are expressed as means ± SEM from 2 experiments. *, *P* < 0.05; ***, *P* < 0.005; n.s., not significant.

As shown above, Pba is able to inhibit SARS-CoV-2 and MERS-CoV infection under white light exposure in cell culture. To determine if Pba could be used *in vivo*, its antiviral activity was tested in a preclinical model, the human primary airway epithelial cells. These cells, Mucilair, are primary bronchial epithelial cells reconstituted in a three-dimensional structure to mimic bronchial epithelium with an air-liquid interface. Mucilair cells were inoculated with SARS-CoV-2 or MERS-CoV in the presence of Pba at 0.25 or 2.5 μM. Remdesivir at 5 μM was used as a positive control. At 72 h postinoculation, viral titers were determined and viral RNA levels were quantified. Viral RNA levels of SARS-CoV-2 and MERS-CoV were significantly decreased in cells in the presence of Pba at 0.25 and 2.5 μM, respectively (Fig. 8A). Similarly, viral titers of both viruses were decreased more than 1 log_10_ in the presence of Pba at 2.5 μM (Fig. 8B). In parallel, the toxicity of Pba was measured in Mucilair by measuring lactate dehydrogenase (LDH) secretion and transepithelial electrical resistance. Cells were incubated with the molecule under the same condition as the infection assay. Cells treated with lysis buffer were used as a control. No toxicity was observed up to 72 h in the cells treated with Pba compared to the control (Fig. 8C and D). In cells treated with lysis buffer, no LDH secretion was observed at 48 h and 72 h because the cells were dead. Taken together, these results confirm the antiviral activity of Pba against highly pathogenic human CoVs and its potential activity *in vivo*.

## DISCUSSION

By screening plant extracts for their antiviral activity against coronaviruses, the present study identified Pba as the active antiviral compound in the crude methanol extract from *M. opositifolius* after bioguided fractionation. It was demonstrated that Pba is active against various human CoVs, including SARS-CoV-2, HCoV-229E, and MERS-CoV, as well as other enveloped viruses, including HCV, SINV, and YFV, and various pseudotyped particles. Furthermore, we characterized Pba as a broad-spectrum antiviral photosensitizer causing PDI of all tested enveloped viruses by production of singlet oxygen species that most probably increase the rigidity of the lipid bilayer of the viral envelope.

Pba is a product of chlorophyll breakdown that is abundantly present in various plants (such as spinach) and marine algae. In general, the chlorophyll content in plants can vary and depends on the season, the part of the plant, the maturity of the organs, and many other factors (29, 30). Chlorophyll is degraded into Pba by chlorophyllase, and some plants with high chlorophyllase content may contain more Pba (31, 32). Pba is well documented for its potential as an anticancer agent in photodynamic therapy (PDT). It is known to have a low toxicity, to selectively accumulate in tumors, and to have a high adsorption at 665 nm (33, 34).

For many years, photosensitizers have mainly been used as antitumor therapy, for which many photosensitizers have already been proven to be clinically safe, and some are currently approved for use in humans (35). Although reports on the PDI of viruses go back to 1960, where it was shown that some photosensitive dyes, such as methylene blue, had an antiviral effect, it is only in the last decades that photosensitizers have gained considerable interest as antimicrobial (bacteria, fungi, and viruses) agents due to their strong antimicrobial effects and low toxicity in normal tissue (36, 37). A major advantage of those molecules is that, due to their direct damaging effect on the microorganism, they are insensitive to the onset of resistance of the microorganism against the compound, the latter being a major problem in today’s research on antivirals and antibiotics. With the present study, we add Pba to the list of photosensitizers with considerable antiviral effects, at least against enveloped viruses. This antiviral effect is not new, since the antiviral activity of Pba has already been demonstrated before against several enveloped viruses, including HCV, influenza A, herpes simplex-2 virus, and HIV-1 (26–28). Zhang et al. (28) also showed a direct action on the viral particle, which is in line with our results. In our study, we show that the coronavirus’ envelope remains intact after treatment with Pba up to 10 μM for 30 min in the light, that Pba treatment does not affect attachment, and that the virus-cell fusion is impaired probably due to the rigidification of the virus membrane upon light exposure. In addition, there seemed to be a slight antiviral effect at high concentrations (more than 10 μM) even under dark conditions. In contrast to our results, some papers show that Pba has a virucidal activity by damaging the virus envelope, thereby inhibiting attachment (28). For HCV and HIV-1, Pba and chlorophyll *a* derivatives were applied after virus infection and were shown to act on replication, corresponding to a mode of action different than the one reported in our study. Furthermore, none of those reports showed a mechanism of action dependent on light exposure and, hence, did not show that the antiviral effect is mediated by PDI of the particle. It is important to note that under usual experimental conditions, cell cultures were not protected from light; thus, it is likely that the vast majority of the reports on antiviral activity of Pba were performed under light exposure. Due to the lack of control conditions in the dark or information on the time of (unnoticed) light exposure in the above-mentioned reports on Pba, it is very hard to compare our data with the literature at this moment. More in-depth studies will be needed to understand and compare the action of Pba on different viruses under both light and dark conditions.

It is interesting that Pba (or highly related compounds such as pyroPba) has been isolated from different plant species and different organisms, including marine algae (38), rendering Pba a very attractive antiviral due to its high availability. Moreover, it has been postulated that Pba can transport a metal ion like zinc inside the cell and that zinc can inhibit viral replication (39), which might be another advantage to using these molecules as antivirals. In our study, it was confirmed that neither natural nor commercial Pba used in our experiments contained a metal ion, so future studies should be performed with zinc Pba to demonstrate this hypothesis.

Here, we show that Pba inhibits virus-cell fusion, probably by targeting and photodynamically damaging the viral membrane. With the help of cryo-EM, we demonstrated that the treatment of virions with Pba and exposed to the light did not affect their shape despite an osmotic shock. This is an indirect demonstration of an increased rigidity of the viral envelope upon Pba treatment. This feature was already demonstrated using a biophysical approach with lipophilic photosensitizers with antiviral activity (24, 40). It was postulated that the increased rigidity impairs membrane bending, required for viral fusion (41). Contrary to the cell membrane, the viral envelope is not able to undergo regeneration, which renders the PDI virus specific and insensitive to the onset of resistance. Very recently, Tummino et al. suggested that many repurposed drugs targeted as SARS-CoV-2 antivirals are cationic amphiphilic drugs (CAD), which induce phospholipolysis (42). CAD are host target antivirals that have an action on cell phospholipids, provoke phospholipidosis, and disrupt the double membrane vesicles necessary for viral replication. The mechanism of action of the CAD is different from the one of Pba. Pba is a virus target antiviral, and it was never reported to have phospholipidosis activity. Pba most probably induces oxidation of the viral envelope phospholipids and, as was shown by cryo-EM in our study, does not induce phospholipidosis.

In the present study, several other compounds structurally related to Pba and to other known photosensitizers were also screened for their anticoronavirus activity to find out whether other compounds would have a more potent effect. Several of those compounds, including pyroPba, chlorin e6, HPPH, *N*-methyl protoporphyrin IX, *N*-methyl mesoporphyrin IX, and Zn-protoporphyrin IX, had a light-dependent antiviral effect, but only pyroPba turned out to be more active than Pba. However, pyroPba was also more toxic; hence, the final selectivity index was not higher than that of Pba. Several chlorophyll derivates, like pyroPba and pheophytin a, have already been demonstrated to be active against IAV, herpes simplex 2 virus, HCV, RSV (respiratory syncytial virus), and SARS-CoV-2 (26, 27, 43). The authors studied the mechanism of action of pyroPba against IAV and showed that the molecule targets the membrane of the virus and not the surface glycoproteins, a mechanism that is consistent with the one that we observed for Pba in our study. A requirement for photoactivation of pyroPba was not investigated or mentioned by Chen et al. (43). Chlorophyllides, chlorophyll precursors, are another class of chlorophyll derivates that have been shown to have antiviral activity against several viruses, including hepatitis B virus (44–46). Other porphyrins have already been described for their antiviral activity (25, 47). Interestingly, three photosensitizers that have been shown to be active against VSV, including *N*-methyl protoporphyrin IX, *N*-methyl mesoporphyrin IX, and Zn-protoporphyrin IX, were also identified in our screen (48). The authors clearly demonstrated that these compounds inactivated VSV after photoactivation via singlet oxygen release. We did not demonstrate the mechanism of action of these three molecules against HCoV-229E, but we demonstrated that they are active after photoactivation. Very recently, protoporphyrin IX and verteporfin were identified as inhibitors of SARS-CoV-2 (49, 50). Both studies showed that protoporphyrin IX is active at an early step of infection, probably the entry step. Gu et al. postulated that the interaction of the compounds with ACE2 impairs the interaction of the virus with its receptor (49). Lu et al. showed that protoporphyrin IX is active against several enveloped viruses but that the activity of protoporphyrin IX against IAV is not dependent on light activation (50). Chlorin e6 is one of the most active compounds against HCoV-229E identified in this study. The antiviral activity of chlorin e6 against enveloped viruses such as HBV (hepatitis B virus), HCV, HIV, DENV (dengue virus), MARV (Marburg virus), TCRV (tacaribe virus), and JUNV (Junin virus) has been demonstrated already (46). Interestingly, the authors also showed that the molecule is inactive against nonenveloped viruses, suggesting that it targets the viral envelope.

As mentioned above, many other photosensitizers have been studied for their antiviral activity (25, 41), and for some of them, the PDI was clearly demonstrated as the mechanism of action (41, 51). In light of the current SARS-CoV-2 pandemic, photosensitizers have received renewed attention as antiviral tools to face this pandemic, and the use of those substances for the treatment of COVID-19 or the inactivation of SARS-CoV-2 on surfaces or in water has been postulated (52, 53). Pba might have some advantages above the already-described photosensitizers, as it is a highly available natural product and active under normal light conditions. Importantly, it does not require a very specific wavelength-dependent illumination treatment, at least not when applied on surfaces/mucosae exposed to environmental light, with an absorption at 667 nm. However, the light dependency of such molecules might render their application as therapeutic agents for internal organs (such as lungs for SARS-CoV-2) more challenging. Indeed, additional illumination will make its application as real therapy more complex, though not impossible, because PDT is already used for the treatment of lung cancer (54). Efforts should be made for the development of a specific device allowing PDT for COVID patients. Nonetheless, we believe that broad-spectrum, low-toxicity, non-resistance-inducing molecules such as Pba can certainly prove their value to reduce environment-to-person and person-to-person transmission of microorganisms when applied as, e.g., a spray for decontamination of surfaces or when formulated for topical application in nose and mouth. Very recently, a study describing such topical application of synthetic SARS-CoV-2 fusion inhibitor has demonstrated that the topical treatment of upper respiratory tract infections might prove its value in reducing virus transmission, particularly in cases where many people gather (55). In contrast to the SARS-CoV-2 fusion inhibitor described by de Vries et al. (55), Pba is a widely commercially available natural molecule with broad-spectrum activity against many enveloped viruses. Therefore, one should explore whether it can exert similar effects upon topical administration to the nose or oral cavity. If so, Pba might help to make people less susceptible to and/or less contagious upon upper respiratory infections with enveloped viruses, many of them causing seasonal outbreaks of respiratory disease such as common colds and flu.

Given that (i) onset of resistance to this product is very unlikely, (ii) the activity of the compound is not dependent of envelope variants, (iii) coronaviruses and other enveloped viruses can cause major problems in animals, and (iv) there is a potential risk for virus transmission from those animals to humans, formulating Pba in such a way that veterinary medicine and facilities with large numbers of animals can benefit from the strong antiviral properties that this molecule might have in the environment (decontamination of air, water, and surfaces) should be explored.

## MATERIALS AND METHODS

### Chemicals.

Dulbecco’s modified Eagle’s medium (DMEM), Opti-MEM, phosphate-buffered saline (PBS), and 4′,6-diamidino-2-phenylindole (DAPI) were purchased from Life Technologies. Goat and fetal bovine sera (FBS) were obtained from Eurobio. Pheophorbide a (Pba), >90% pure, pyroPba, chlorin e6, HPPH, *N*-methyl protoporphyrin IX, *N*-methyl mesoporphyrin IX, and Zn protoporphyrin IX were from Cayman Chemicals (Merck Chemicals, Darmstadt, Germany). Remdesivir (GS-5734) was from Selleck Chemicals (Houston TX). Mowiol 4-88 was obtained from Calbiochem. Rose Bengale, Trolox, and other chemicals were from Sigma (St. Louis, MO). Stocks of compounds were resuspended in dimethyl sulfoxide (DMSO) at 50 mM. Plant extracts were resuspended in DMSO at 25 mg/ml.

### Antibodies.

Mouse anti-HCV E1 monoclonal antibody (MAb) A4 (56) and mouse anti-YFV E MAb 2D12 (anti-E; ATCC CRL-1689) were produced *in vitro* by using a MiniPerm apparatus (Heraeus). Mouse anti-double-stranded RNA MAb (clone J2) was obtained from Scicons. Mouse anti-SARS-CoV-2 spike protein MAb were obtained from GeneTex. Polyclonal rabbit anti-SARS-CoV-2 nucleocapsid antibodies were from Novus. Cyanine 3-conjugated goat anti-mouse IgG and horseradish peroxidase (HRP)-labeled goat-anti-rabbit IgG antibodies were from Jackson Immunoresearch.

### Cells and culture conditions.

Huh-7, Vero-81 (ATCC number CCL-81), and Vero-E6 cells were grown in DMEM with GlutaMAX-I and 10% FBS in an incubator at 37°C with 5% CO_2_. Vero-81 cells were subcloned to obtain a better overall infection rate. The Mucilair primary human bronchial epithelial cells were from Epithelix (Geneva, Switzerland) and maintained in Mucilair culture medium (Epithelix) as recommended by the manufacturer.

### Plant collection and extraction.

The 15 plants were collected in the Bafing region (Northwest Côte d’Ivoire, Touba Department). They were authenticated at the Centre National de Floristique (CNF), University of Félix Houphouët Boigny de Cocody (Abidjan), where voucher specimens were deposited in a herbarium. The *M. oppositifolius* voucher number is UCJ006172. Plants were cleaned and air dried at constant temperature (26°C) for 1 to 2 weeks at the Nangui Abrogoua University (Abidjan). They were then powdered and stored in the dark until extractions. For each plant, 20 g of dried powder was mixed with 100 ml methanol for 24 h. After filtration, the grounds were extracted again twice in the same way. The 3 resulting filtrates were combined and dried under vacuum at 40°C. These extracts were then dissolved in DMSO for antiviral assays.

### Bioguided fractionation of Mo extract and Pba identification.

For *Mallotus oppositifolius*, three other solvents were used to extract more compounds from these plant leaves: methylene chloride (MC) for the first extraction of the dried leaves, methanol to extract the first ground, and ethanol-water (50:50) to extract the second ground. The corresponding extracts were tested against HCoV-229E-Luc. Since the MC extract was the most active, it was fractionated by chromatography (CPC), leading to 10 fractions (F1 to F10) that were tested again. F7 was selected for further fractionation by another round of chromatography (HPLC) that led to 9 partitions (7.1 to 7.9). For partition 7.7 (the most active against HCoV-229E), purity and identity were determined by UPLC-MS and NMR (more details are in the supplemental material).

### Viruses.

HCoV-229E strain VR-740 (ATCC) and recombinant HCoV-229E-Luc (kind gift of V. Thiel) were used (57). SARS-CoV-2 (isolate SARS-CoV-2/human/FRA/Lille_Vero-81-TMPRSS2/2020; NCBI MW575140) was propagated on Vero-81-TMPRSS2 cells. MERS-CoV was recovered by transfecting the infectious clone of MERS-CoV-EMC12 (kindly provided by Luis Enjuanes) in Huh-7 cells. A cell culture-adapted strain (JFH1-CSN6A4) of HCV was produced as previously described (58). A recombinant Sindbis virus (SINV) expressing HCV E1 glycoprotein was employed as previously described (59). YFV strain 17D was obtained from Philippe Desprès (Institut Pasteur de Paris, France). Coxsackievirus B4 strain E2 (CVB4) was provided by Didier Hober (Université de Lille, France).

### Cell toxicity assay.

A total of 6 × 10^4^ Huh-7, Vero-E6, and Vero-81 cells were seeded in 96-well plates and incubated for 16 h at 37°C in a 5% CO_2_ incubator. The cells were then treated with increasing concentrations of the compound of interest. One hour after inoculation, cells were taken out of the incubator to be exposed to the white light of a biosafety cabinet (BSC) for 10 min, after which cells were further incubated in the dark at 37°C and 5% CO_2_ for 23 h. The BSC’s light source lamp consists of one fluorescent tube of 36 W, 3,350 lumen, white light. An MTS [3-(4,5-dimethylthiazol-2-yl)-5-(3-carboxymethoxyphenyl)-2-(4-sulfophenyl)-2H-tetrazolium]-based viability assay (Cell Titer 96 aqueous nonradioactive cell proliferation assay; Promega) was performed as recommended by the manufacturer. The absorbance of formazan at 490 nm was detected using a plate reader (ELX 808 Bio-Tek Instruments Inc.). Each measure was performed in triplicate, and each experiment was repeated at least 3 times.

### Micilair toxicity assays. (i) LDH secretion assay.

Mucilair cells, in three wells per condition, were incubated with Pba in 100 μl Mucilair culture medium or 50 μl of lysis solution (cytotoxicity LDH assay kit-WST; Dojindo) at the apical surface for 1 h. Pba or lysis solution was removed, and the cells were placed in the incubator for 72 h. At 24 h, 48 h, and 72 h, LDH secretion was measured in the basolateral medium according to the manufacturer’s instructions by recording the absorbance of WST-formazan at 490 nm. The results are expressed relative to the LDH secretion values obtained in the lysed wells, for which a value of 100% toxicity was attributed.

### (ii) Transepithelial electrical resistance.

Mucilair cells were incubated with Pba and lysis solution as described above. Transepithelial electrical resistance was measured using a Volt/Ohm-meter and electrode (Millicell ERS2; Millipore) at 24 h, 48 h, and 72 h; 200 μl of prewarmed Mucilair culture medium was added at the apical surface prior to resistance measurement. Data are expressed relative to control untreated cells.

### HCoV-229E infection inhibition assays. (i) Luciferase assay.

HCoV-229E-Luc was first mixed with the crude extracts or the compounds at the appropriate concentrations for 10 min. Huh-7 cells and Huh-7-TMPRSS2 cells were inoculated with HCoV-229E-Luc at a multiplicity of infection (MOI) of 0.5 in a final volume of 50 μl for 1 h at 37°C in the presence of the plant crude extracts or the different compounds. The virus was removed and replaced with culture medium containing the extracts or the different compounds for 6 h at 37°C. Cells were lysed in 20 μl of Renilla lysis buffer (Promega, Madison, USA), and luciferase activity was quantified in a Tristar LB 941 luminometer (Berthold Technologies, Bad Wildbad, Germany) using a Renilla luciferase assay system (Promega) as recommended by the manufacturer.

This experiment was either performed under white light exposure, in which the virus and the compounds were exposed to the light of the BSC lamp. To maximize light exposure, the tubes were laid flat on the bench of the BSC. For dark conditions, the light of the BSC and the room was shut down and all the tubes and plates were covered with foil paper.

### (ii) HCoV-229E titers.

Huh-7 and Huh-7-TMPRSS2 cells seeded in 24-well plates were inoculated with HCoV-229E at an MOI of 0.5 in the presence of Pba at different concentrations for 1 h at 37°C. The inoculum was removed and replaced with culture medium containing Pba, and the cells were incubated at 37°C for 8 h (for TMPRSS2 condition) or 10 h (without TMPRSS2). Supernatants were collected and serial dilutions were performed and used to infect naive Huh-7 cells in 96-well plates. Six days after infection, cytopathic effect was determined in each well to calculate 50% tissue culture infectious dose (TCID_50_) titers by using the Reed and Muench method.

### SARS-CoV-2 and MERS-CoV infection inhibition assays.

Vero-E6 and Huh-7 cells seeded in 24-well plates 24 h before inoculation were inoculated with SARS-CoV-2 and MERS-CoV, respectively, at an MOI of 0.3 in the presence of Pba at different concentrations for 1 h at 37°C. The inoculum was removed by 3 washings with DMEM, and fresh medium containing different Pba concentrations was added for 16 h at 37°C. Cell supernatants were collected and the amount of infectious virus was determined by infectivity titration. Therefore, Vero-E6 (SARS-CoV-2) and Huh-7 (MERS-CoV) cells, seeded in 96-well plates, were inoculated with 100 μl of 1/10 serially diluted supernatants (ranging from 10^−1^ to 10^−8^). Cells were incubated with the virus dilutions for 5 days at 37°C and 5% CO_2_. The TCID_50_ then was determined by assessing the cytopathic effect in each well by light microscopy, and the 50% endpoint was calculated according to the method of Reed and Muench.

### Time-of-addition assay.

To determine at which stage of the replication cycle Pba executed its effect, a time-of-addition assay was performed to which 1 μM Pba (and 10 μM chloroquine as a control for SARS-CoV-2) was added at different time points before (referred to as the condition pretreatment cells), during (referred to as the condition inoculation), or after inoculation. For the latter condition, Pba and chloroquine were not added before and during inoculation but only directly after removal of the inoculum (referred to as the condition postinfection [p.i.]–end) or from 1 h or 2 h after removal of the inoculum onwards (referred to as the condition 1 h p.i.–end and 2 h p.i.–end, respectively) and were left in the medium for the rest of the incubation time (i.e., until 6 h p.i. for HCoV-229E and 16 h p.i. for SARS-CoV-2). For this experiment, Huh-7-TMPRSS2 or Vero-81 cells were inoculated with HCoV-229E-Luc or SARS-CoV-2 at an MOI of 0.5 and 0.05, respectively. One hour after inoculation, cells for all conditions were washed 3 times to remove the unbound particles. For HCoV-229E-luc, luciferase activity was quantified as described above. For SARS-CoV-2, cells were washed once with PBS and lysed in 200 μl of nonreducing 2× Laemmli loading buffer. Lysates were incubated at 95°C for 30 min to inactivate the virus, and lysates were kept at −20°C until Western blot analysis (see below). For each time point, DMSO was taken as a control, and all experiments were repeated 3 times.

### Western blot detection of the SARS-CoV-2 nucleocapsid expression.

Sixteen hours after inoculation, cells were washed once with PBS and lysed in 200 μl of nonreducing 2× Laemmli loading buffer. Lysates were incubated at 95°C for 30 min to inactivate the virus, and the proteins were subsequently separated on a 12% polyacrylamide gel by SDS-PAGE. Next, proteins were transferred to a nitrocellulose membrane (Amersham), and the membranes were subsequently blocked for 1 h at room temperature in 5% (wt/vol) nonfat dry milk in PBS with 0.1% (vol/vol) Tween 20. Membranes were incubated overnight at 4°C with polyclonal rabbit anti-SARS-CoV-2 nucleocapsid antibodies in 5% (wt/vol) nonfat dry milk in PBS with 0.1% (vol/vol) Tween 20. After being washed 3 times with PBS with 0.1% (vol/vol) Tween 20, membranes were incubated for 1 h at room temperature with HRP-labeled goat-anti rabbit IgG antibodies, after which membranes were washed 3 times. N proteins were visualized by enhanced chemiluminescence (Pierce ECL, ThermoFisher Scientific). Quantification was performed by using Image J and its gel quantification function.

### Infection assay with other viruses.

Vero (YFV, SINV, and CBV4) or Huh-7 (HCV) cells grown on glass coverslips were infected with viral stocks diluted so as to obtain 20 to 40% infected cells under control conditions. The cells were fixed at a time that allowed for clear detection of infected cells versus noninfected cells and avoided the detection of reinfection events, limiting the analysis to a single round of infection (30 h p.i. for HCV, 20 h p.i. for YFV, 6 h p.i. for SINV, and 4 h p.i. for CVB4). The cells were fixed for 20 min with 3% PFA. They were then rinsed with PBS and processed for immunofluorescence as previously described (60) using primary mouse antibodies specific to HCV E1 (for both HCV and SINV), YFV E, or double-stranded RNA (for CVB4), followed by a cyanine-3-conjugated goat anti-mouse IgG secondary antibody for the detection of infected cells. Nuclei were stained with DAPI. Coverslips were mounted on microscope slides in Mowiol 4-88-containing medium. Images were acquired on an Evos M5000 imaging system (Thermo Fisher Scientific) equipped with light cubes for DAPI, red fluorescent protein (RFP), and a 10× objective. For each coverslip, a series of six 8-bit images of randomly picked areas were recorded. Cells labeled with anti-virus MAbs were counted as infected cells. The total number of cells was obtained from DAPI-labeled nuclei. Infected cells and nuclei were automatically counted using macros written in ImageJ. Infections were scored as the ratio of infected over total cells. The data are presented as the percentage of infection relative to the control condition.

### Effect of Pba on pseudotyped virion entry.

Particles pseudotyped with either SARS-CoV-2 S (SARS-2pp), MERS-CoV S proteins (MERSpp), HCoV-229E-S (HCoV-229Epp), genotype 2a HCV envelope proteins (HCVpp), or the G envelope glycoprotein of vesicular stomatitis virus (VSV-Gpp) were produced as previously described (22, 61). Pseudotyped virions were pretreated with Pba for 30 min at room temperature under the BSC’s light or covered in foil and then used to inoculate Huh-7 cells in 96-well plates for 3 h. The inoculum was removed and cells were further incubated with culture medium for 45 h. Cells were lysed and luciferase activity was detected by using a Luciferase assay kit (Promega) and light emission measured by using a Tristar LB 941 luminometer (Berthold Technologies).

### White light exposure kinetics.

HCoV-229E-Luc or SARS-CoV-2 were pretreated with Pba at room temperature and exposed to the BSC’s white fluorescent light for different periods of time. To maximize light exposure, tubes were laid flat under the BSC’s light. Next, infection was quantified for each virus as described previously.

### Fusion assay.

Cells were preincubated for 30 min in the presence of 25 mM NH_4_Cl at 37°C to inhibit virus entry through the endosomal route and then were transferred to ice. In the meantime, the virus was preincubated under light with Pba and 25 mM NH_4_Cl for 10 min and then allowed to bind to the cells at 4°C for 1 h in DMEM containing 0.2% bovine serum albumin (BSA), 20 mM HEPES, and 25 mM NH_4_Cl. Cells were then warmed by addition of DMEM containing 3 μg/ml trypsin, 0.2% BSA, 20 mM HEPES, and 25 mM NH_4_Cl and were incubated for 5 min in a water bath at 37°C. The cells were rinsed and further incubated for 30 min in culture medium containing 25 mM NH_4_Cl, and then the medium was replaced by normal culture medium. Seven hours after inoculation, luciferase activity was detected by using a Renilla Luciferase assay kit (Promega).

### Cell-cell fusion assay by transient expression of the SARS-CoV-2 spike protein.

Vero-81 cells were seeded on coverslips in 24 wells 16 h before transfection. Cells were transfected with 250 ng of a pCDNA3.1(+) vector encoding the SARS-CoV-2 spike protein using the TransIT-LT1 transfection reagent (Mirus Bio). Six hours posttransfection (p.t.), transfection medium was replaced by normal medium containing 1 μM Pba or DMSO. Twenty-four hours p.t., cells were fixed with 3% paraformaldehyde in PBS for 20 min at room temperature and syncytia were visualized by immunofluorescence by incubating the cells with a monoclonal anti-SARS-CoV-2-spike antibody in 10% normal goat serum, followed by incubation with cyanine-3-conjugated goat anti-mouse IgG antibodies. Nuclei were visualized with 1 μg/ml DAPI, and coverslips were mounted in Mowiol mounting medium. Pictures were obtained with an Evos M5000 imaging system (Thermo Fisher Scientific).

### Attachment assay.

Huh-7-TMPRSS2 cells seeded in 24-well plates were inoculated with HCoV-229E at an MOI of 4 on ice in the presence of 4.1 or 8.2 μM Pba under the light of the BSC. One hour after inoculation, cells were washed 3 times with cold PBS and lysed using LBP lysis buffer for RNA extraction by following the manufacturer’s instructions (NucleoSpin RNA plus extraction kit; Macherey-Nagel). Reverse transcription was then performed on 10 μl of RNA using a high-capacity cDNA reverse transcription kit (Applied Biosystems). Three microliters of cDNA was used for real-time reverse-transcription PCR (qRT-PCR) assay using specific primers and probe targeting the N gene (forward primer, 5′-TTCCGACGTGCTCGAACTTT-3′; reverse primer, 5′-CCAACACGGTTGTGACAGTGA-3′; and probe, 5′-6FAM-TCCTGAGGTCAATGCA-3′) and subjected to qPCR amplification with TaqMan master mix.

### Quencher assay.

HCoV-229E-Luc was mixed with 10 mM Trolox or NaN_3_, after which 0.5 or 1 μM Pba was added and the mixture was exposed to light for 10 min. The mixture was used to inoculate Huh-7-TMPRSS2 cells for 1 h. Inoculum was replaced with DMEM, and cells were kept in the dark at 37°C 5% CO_2_ for 7 h and then lysed to quantify luciferase activity as described above.

### Cryo-EM.

HCoV-229E was produced by inoculating a confluent Huh-7 T75 flask at an MOI of 0.008 in DMEM supplemented with 5% FBS and kept at 33°C and 5% CO_2_ for 5 days. Supernatant was harvested and treated with DMSO or 10 μM Pba and further kept in the dark or exposed to light for 30 min. NaCl then was added to a final concentration of 400 mM to induce an osmotic shock. Viruses were fixed in 4% PFA. For cryo-EM experiments of the particles, lacey carbon Formvar 300 mesh copper grids were used after a standard glow-discharged procedure. Plunge freezing was realized using the EM-GP apparatus (Leica). Specimens were observed at −175°C using a cryo holder (626; Gatan) with a ThermoFisher FEI Tecnai F20 electron microscope operating at 200 kV under low-dose conditions. Images were acquired with an Eagle 4k by 4k camera (ThermoFisher FEI).

### Primary airway cell infection quantification.

The air interface of Mucilair (Epithelix) was rinsed with 100 μl of Mucilair culture medium for 10 min 3 times to remove mucosal secretion. The cells were then inoculated at the apical membrane with SARS-CoV-2 or MERS-CoV at an MOI of 0.2 in the presence of compounds for 1 h at 37°C. Inoculum was removed, and the cells were rinsed with PBS. In parallel, compounds were added in the basolateral medium; 72 h p.i., viruses secreted at the apical membrane were collected by adding 200 μl of medium in the apical chamber. Viral titers were determined as described above. In parallel, cells were lysed with lysis buffer from the kit NucleoSpin RNA plus (Macherey-Nagel), and total RNA was extracted by following the manufacturer’s instructions, eluted in a final volume of 60 μl of H_2_O, and quantified.

For SARS-CoV-2, one-step qPCR assay was performed using 5 μl of RNA and Takyon Low rox one-step RT probe master mix (Eurogentec) and specific primers and probe targeting the E gene (forward primer, 5′-ACAGGTACGTTAATAGTTAATAGCGT-3′; reverse primer, 5′-ATATTGCAGCAGTACGCACACA-3′; and probe, FAM-ACACTAGCCATC-CTTACTGCGCTTCG-MGB).

For MERS-CoV and RPLP0 reference genes, 10 μM RNA was used for cDNA synthesis using a high-capacity cDNA reverse transcription kit (Applied Biosystems); 3 μl of cDNA was used for qRT-PCR assay using specific probes. For MERS-CoV, the following primers and probe targeting N gene were used: forward primer, 5′-GGGTGTACCTCTTAATGCCAATTC-3′; reverse primer, 5′-TCTGTCCTGTCTCCGCCAAT-3′; and probe, 5′-FAM-ACCCCTGCGCAAAATGCTGGG-MGBNFQ-3′. Samples were subjected to qPCR amplification with TaqMan Master mix. For RPLP0, TaqMan gene expression assay (Life Technologies) was used according to the manufacturer’s instructions. SARS-CoV-2 E and MERS-CoV N gene expression was quantified relative to RPLP0 using the ΔΔ*C_T_* method. A value of 1 was arbitrarily assigned to infected cells without compound.

### Statistical analysis and IC_50_ and CC_50_ calculation.

Values were graphed and IC_50_ calculated by nonlinear regression curve fitting with variable slopes constraining the top to 100% and the bottom to 0%, using GraphPad Prism software. Kruskal-Wallis nonparametric test followed by a Dunn’s multicomparison *post hoc* test with a confidence interval of 95% was used to identify individual difference between treatments. *P* values of <0.05 were considered significantly different from the control.

## References

[1] Zhu N, Zhang D, Wang W, Li X, Yang B, Song J, Zhao X, Huang B, Shi W, Lu R, Niu P, Zhan F, Ma X, Wang D, Xu W, Wu G, Gao GF, Tan W, China Novel Coronavirus Investigating and Research Team. 2020. A novel coronavirus from patients with pneumonia in China, 2019. N Engl J Med 382:727–733. 10.1056/NEJMoa2001017. 31978945 PMC7092803

[2] Huang C, Wang Y, Li X, Ren L, Zhao J, Hu Y, Zhang L, Fan G, Xu J, Gu X, Cheng Z, Yu T, Xia J, Wei Y, Wu W, Xie X, Yin W, Li H, Liu M, Xiao Y, Gao H, Guo L, Xie J, Wang G, Jiang R, Gao Z, Jin Q, Wang J, Cao B. 2020. Clinical features of patients infected with 2019 novel coronavirus in Wuhan, China. Lancet 395:497–506. 10.1016/S0140-6736(20)30183-5. 31986264 PMC7159299

[3] Guan W, Ni Z, Hu Y, Liang W, Ou C, He J, Liu L, Shan H, Lei C, Hui DSC, Du B, Li L, Zeng G, Yuen K-Y, Chen R, Tang C, Wang T, Chen P, Xiang J, Li S, Wang J, Liang Z, Peng Y, Wei L, Liu Y, Hu Y, Peng P, Wang J, Liu J, Chen Z, Li G, Zheng Z, Qiu S, Luo J, Ye C, Zhu S, Zhong N, China Medical Treatment Expert Group for Covid-19. 2020. Clinical characteristics of coronavirus disease 2019 in China. N Engl J Med 382:1708–1720. 10.1056/NEJMoa2002032. 32109013 PMC7092819

[4] Cui J, Li F, Shi Z-L. 2019. Origin and evolution of pathogenic coronaviruses. Nat Rev Microbiol 17:181–192. 10.1038/s41579-018-0118-9. 30531947 PMC7097006

[5] Chen Y, Liu Q, Guo D. 2020. Emerging coronaviruses: genome structure, replication, and pathogenesis. J Med Virol 92:418–423. 10.1002/jmv.25681. 31967327 PMC7167049

[6] Riva L, Yuan S, Yin X, Martin-Sancho L, Matsunaga N, Pache L, Burgstaller-Muehlbacher S, De Jesus PD, Teriete P, Hull MV, Chang MW, Chan JF-W, Cao J, Poon VK-M, Herbert KM, Cheng K, Nguyen T-TH, Rubanov A, Pu Y, Nguyen C, Choi A, Rathnasinghe R, Schotsaert M, Miorin L, Dejosez M, Zwaka TP, Sit K-Y, Martinez-Sobrido L, Liu W-C, White KM, Chapman ME, Lendy EK, Glynne RJ, Albrecht R, Ruppin E, Mesecar AD, Johnson JR, Benner C, Sun R, Schultz PG, Su AI, García-Sastre A, Chatterjee AK, Yuen K-Y, Chanda SK. 2020. Discovery of SARS-CoV-2 antiviral drugs through large-scale compound repurposing. Nature 586:113–119. 10.1038/s41586-020-2577-1. 32707573 PMC7603405

[7] Touret F, Gilles M, Barral K, Nougairède A, van Helden J, Decroly E, de Lamballerie X, Coutard B. 2020. In vitro screening of a FDA approved chemical library reveals potential inhibitors of SARS-CoV-2 replication. Sci Rep 10:13093. 10.1038/s41598-020-70143-6. 32753646 PMC7403393

[8] Belouzard S, Machelart A, Sencio V, Vausselin T, Hoffmann E, Deboosere N, Rouillé Y, Desmarets L, Séron K, Danneels A, Robil C, Belloy L, Moreau C, Piveteau C, Biela A, Vandeputte A, Heumel S, Deruyter L, Dumont J, Leroux F, Engelmann I, Alidjinou EK, Hober D, Brodin P, Beghyn T, Trottein F, Déprez B, Dubuisson J. 2021. Large scale screening discovers clofoctol as an inhibitor of SARS-CoV-2 replication that reduces COVID-19-like pathology. bioRxiv 10.1101/2021.06.30.450483.

[9] Jeong GU, Song H, Yoon GY, Kim D, Kwon Y-C. 2020. Therapeutic strategies against COVID-19 and structural characterization of SARS-CoV-2: a review. Front Microbiol 11:1723. 10.3389/fmicb.2020.01723. 32765482 PMC7381222

[10] White KM, Rosales R, Yildiz S, Kehrer T, Miorin L, Moreno E, Jangra S, Uccellini MB, Rathnasinghe R, Coughlan L, Martinez-Romero C, Batra J, Rojc A, Bouhaddou M, Fabius JM, Obernier K, Dejosez M, Guillén MJ, Losada A, Avilés P, Schotsaert M, Zwaka T, Vignuzzi M, Shokat KM, Krogan NJ, García-Sastre A. 2021. Plitidepsin has potent preclinical efficacy against SARS-CoV-2 by targeting the host protein eEF1A. Science 371:926–931. 10.1126/science.abf4058. 33495306 PMC7963220

[11] Li W, Moore MJ, Vasilieva N, Sui J, Wong SK, Berne MA, Somasundaran M, Sullivan JL, Luzuriaga K, Greenough TC, Choe H, Farzan M. 2003. Angiotensin-converting enzyme 2 is a functional receptor for the SARS coronavirus. Nature 426:450–454. 10.1038/nature02145. 14647384 PMC7095016

[12] Hoffmann M, Kleine-Weber H, Schroeder S, Krüger N, Herrler T, Erichsen S, Schiergens TS, Herrler G, Wu N-H, Nitsche A, Müller MA, Drosten C, Pöhlmann S. 2020. SARS-CoV-2 cell entry depends on ACE2 and TMPRSS2 and is blocked by a clinically proven protease inhibitor. Cell 181:271–280. 10.1016/j.cell.2020.02.052. 32142651 PMC7102627

[13] Raj VS, Mou H, Smits SL, Dekkers DHW, Müller MA, Dijkman R, Muth D, Demmers JAA, Zaki A, Fouchier RAM, Thiel V, Drosten C, Rottier PJM, Osterhaus ADME, Bosch BJ, Haagmans BL. 2013. Dipeptidyl peptidase 4 is a functional receptor for the emerging human coronavirus-EMC. Nature 495:251–254. 10.1038/nature12005. 23486063 PMC7095326

[14] Yeager CL, Ashmun RA, Williams RK, Cardellichio CB, Shapiro LH, Look AT, Holmes KV. 1992. Human aminopeptidase N is a receptor for human coronavirus 229E. 6377. Nature 357:420–422. 10.1038/357420a0. 1350662 PMC7095410

[15] Matsuyama S, Nagata N, Shirato K, Kawase M, Takeda M, Taguchi F. 2010. Efficient activation of the severe acute respiratory syndrome coronavirus spike protein by the transmembrane protease TMPRSS2. J Virol 84:12658–12664. 10.1128/JVI.01542-10. 20926566 PMC3004351

[16] Shirato K, Kawase M, Matsuyama S. 2013. Middle East respiratory syndrome coronavirus infection mediated by the transmembrane serine protease TMPRSS2. J Virol 87:12552–12561. 10.1128/JVI.01890-13. 24027332 PMC3838146

[17] Belouzard S, Millet JK, Licitra BN, Whittaker GR. 2012. Mechanisms of coronavirus cell entry mediated by the viral spike protein. Viruses 4:1011–1033. 10.3390/v4061011. 22816037 PMC3397359

[18] Huang F, Li Y, Leung EL-H, Liu X, Liu K, Wang Q, Lan Y, Li X, Yu H, Cui L, Luo H, Luo L. 2020. A review of therapeutic agents and Chinese herbal medicines against SARS-COV-2 (COVID-19). Pharmacol Res 158:104929. 10.1016/j.phrs.2020.104929. 32442720 PMC7237953

[19] Mani JS, Johnson JB, Steel JC, Broszczak DA, Neilsen PM, Walsh KB, Naiker M. 2020. Natural product-derived phytochemicals as potential agents against coronaviruses: a review. Virus Res 284:197989. 10.1016/j.virusres.2020.197989. 32360300 PMC7190535

[20] Bertram S, Dijkman R, Habjan M, Heurich A, Gierer S, Glowacka I, Welsch K, Winkler M, Schneider H, Hofmann-Winkler H, Thiel V, Pöhlmann S. 2013. TMPRSS2 activates the human coronavirus 229E for cathepsin-independent host cell entry and is expressed in viral target cells in the respiratory epithelium. J Virol 87:6150–6160. 10.1128/JVI.03372-12. 23536651 PMC3648130

[21] Iwata-Yoshikawa N, Okamura T, Shimizu Y, Hasegawa H, Takeda M, Nagata N. 2019. TMPRSS2 contributes to virus spread and immunopathology in the airways of murine models after coronavirus infection. J Virol 93:e01815-18. 10.1128/JVI.01815-18. 30626688 PMC6401451

[22] Belouzard S, Chu VC, Whittaker GR. 2009. Activation of the SARS coronavirus spike protein via sequential proteolytic cleavage at two distinct sites. Proc Natl Acad Sci USA 106:5871–5876. 10.1073/pnas.0809524106. 19321428 PMC2660061

[23] Kawase M, Shirato K, Matsuyama S, Taguchi F. 2009. Protease-mediated entry via the endosome of human coronavirus 229E. J Virol 83:712–721. 10.1128/JVI.01933-08. 18971274 PMC2612384

[24] Vigant F, Lee J, Hollmann A, Tanner LB, Akyol Ataman Z, Yun T, Shui G, Aguilar HC, Zhang D, Meriwether D, Roman-Sosa G, Robinson LR, Juelich TL, Buczkowski H, Chou S, Castanho MARB, Wolf MC, Smith JK, Banyard A, Kielian M, Reddy S, Wenk MR, Selke M, Santos NC, Freiberg AN, Jung ME, Lee B. 2013. A mechanistic paradigm for broad-spectrum antivirals that target virus-cell fusion. PLoS Pathog 9:e1003297. 10.1371/journal.ppat.1003297. 23637597 PMC3630091

[25] Costa L, Faustino MAF, Neves M, Cunha A, Almeida A. 2012. Photodynamic inactivation of mammalian viruses and bacteriophages. Viruses 4:1034–1074. 10.3390/v4071034. 22852040 PMC3407894

[26] Ratnoglik SL, Aoki C, Sudarmono P, Komoto M, Deng L, Shoji I, Fuchino H, Kawahara N, Hotta H. 2014. Antiviral activity of extracts from Morinda citrifolia leaves and chlorophyll catabolites, pheophorbide a and pyropheophorbide a, against hepatitis C virus. Microbiol Immunol 58:188–194. 10.1111/1348-0421.12133. 24438164

[27] Bouslama L, Hayashi K, Lee J-B, Ghorbel A, Hayashi T. 2011. Potent virucidal effect of pheophorbide a and pyropheophorbide a on enveloped viruses. J Nat Med 65:229–233. 10.1007/s11418-010-0468-8. 20835849

[28] Zhang H-J, Tan GT, Hoang VD, Hung NV, Cuong NM, Soejarto DD, Pezzuto JM, Fong HHS. 2003. Natural anti-HIV agents. Part IV. Anti-HIV constituents from Vatica cinerea. J Nat Prod 66:263–268. 10.1021/np020379y. 12608862

[29] Yilmaz C, Gökmen V. 2016. Chlorophyll, p 37–41. *In* Caballero B, Finglas PM, Toldrá F (ed), Encyclopedia of food and health. Academic Press, Oxford, United Kingdom.

[30] Solymosi K, Mysliwa-Kurdziel B. 2017. Chlorophylls and their derivatives used in food industry and medicine. Mini Rev Med Chem 17:1194–1222. 10.2174/1389557516666161004161411. 27719668

[31] Humphrey AM. 1980. Chlorophyll. Food Chem 5:57–67. 10.1016/0308-8146(80)90064-3.

[32] Holden M. 1974. Chlorophyll degradation products in leaf protein preparations. J Sci Food Agric 25:1427–1432. 10.1002/jsfa.2740251111. 4437147

[33] Hajri A, Wack S, Meyer C, Smith MK, Leberquier C, Kedinger M, Aprahamian M. 2002. In vitro and in vivo efficacy of photofrin and pheophorbide a, a bacteriochlorin, in photodynamic therapy of colonic cancer cells. Photochem Photobiol 75:140–148. 10.1562/0031-8655(2002)075<0140:IVAIVE>2.0.CO;2. 11883602

[34] Roeder B, Naether D, Lewald T, Braune M, Nowak C, Freyer W. 1990. Photophysical properties and photodynamic activity in vivo of some tetrapyrroles. Biophys Chem 35:303–312. 10.1016/0301-4622(90)80017-2. 2397276

[35] Hamblin MR. 2020. Photodynamic therapy for cancer: what’s past is prologue. Photochem Photobiol 96:506–516. 10.1111/php.13190. 31820824 PMC7282978

[36] Wiehe A, O'Brien JM, Senge MO. 2019. Trends and targets in antiviral phototherapy. Photochem Photobiol Sci 18:2565–2612. 10.1039/c9pp00211a. 31397467

[37] Mariewskaya KA, Tyurin AP, Chistov AA, Korshun VA, Alferova VA, Ustinov AV. 2021. Photosensitizing antivirals. Mol Basel Switz 26:3971. 10.3390/molecules26133971. PMC8271894 34209713

[38] Saide A, Lauritano C, Ianora A. 2020. Pheophorbide a: state of the art. Mar Drugs 18:257. 10.3390/md18050257. PMC7281735 32423035

[39] Clark NF, Taylor-Robinson AW. 2020. COVID-19 therapy: could a chlorophyll derivative promote cellular accumulation of Zn2+ ions to inhibit SARS-CoV-2 RNA synthesis? Front Plant Sci 11:1270. 10.3389/fpls.2020.01270. 32922431 PMC7457044

[40] Hollmann A, Castanho MARB, Lee B, Santos NC. 2014. Singlet oxygen effects on lipid membranes: implications for the mechanism of action of broad-spectrum viral fusion inhibitors. Biochem J 459:161–170. 10.1042/BJ20131058. 24456301

[41] Vigant F, Santos NC, Lee B. 2015. Broad-spectrum antivirals against viral fusion. Nat Rev Microbiol 13:426–437. 10.1038/nrmicro3475. 26075364 PMC4554337

[42] Tummino TA, Rezelj VV, Fischer B, Fischer A, O'Meara MJ, Monel B, Vallet T, White KM, Zhang Z, Alon A, Schadt H, O'Donnell HR, Lyu J, Rosales R, McGovern BL, Rathnasinghe R, Jangra S, Schotsaert M, Galarneau J-R, Krogan NJ, Urban L, Shokat KM, Kruse AC, García-Sastre A, Schwartz O, Moretti F, Vignuzzi M, Pognan F, Shoichet BK. 2021. Drug-induced phospholipidosis confounds drug repurposing for SARS-CoV-2. Science 373:541–547. 10.1126/science.abi4708. 34326236 PMC8501941

[43] Chen D, Lu S, Yang G, Pan X, Fan S, Xie X, Chen Q, Li F, Li Z, Wu S, He J. 2020. The seafood Musculus senhousei shows anti-influenza A virus activity by targeting virion envelope lipids. Biochem Pharmacol 177:113982. 10.1016/j.bcp.2020.113982. 32305436 PMC7162792

[44] Lamontagne J, Mills C, Mao R, Goddard C, Cai D, Guo H, Cuconati A, Block T, Lu X. 2013. Screening and identification of compounds with antiviral activity against hepatitis B virus using a safe compound library and novel real-time immune-absorbance PCR-based high throughput system. Antiviral Res 98:19–26. 10.1016/j.antiviral.2013.02.001. 23415884 PMC3628437

[45] Wang Y-T, Yang C-H, Huang K-S, Shaw J-F. 2021. Chlorophyllides: preparation, purification, and application. Biomolecules 11:1115. 10.3390/biom11081115. 34439782 PMC8392590

[46] Guo H, Pan X, Mao R, Zhang X, Wang L, Lu X, Chang J, Guo J-T, Passic S, Krebs FC, Wigdahl B, Warren TK, Retterer CJ, Bavari S, Xu X, Cuconati A, Block TM. 2011. Alkylated porphyrins have broad antiviral activity against hepadnaviruses, flaviviruses, filoviruses, and arenaviruses. Antimicrob Agents Chemother 55:478–486. 10.1128/AAC.00989-10. 21135183 PMC3028764

[47] Lebedeva NS, A Gubarev Y, O Koifman M, I Koifman O. 2020. The application of porphyrins and their analogues for inactivation of viruses. Mol Basel Switz 25:4368. 10.3390/molecules25194368. PMC7583985 32977525

[48] Cruz-Oliveira C, Almeida AF, Freire JM, Caruso MB, Morando MA, Ferreira VNS, Assunção-Miranda I, Gomes AMO, Castanho MARB, Da Poian AT. 2017. Mechanisms of vesicular stomatitis virus inactivation by protoporphyrin IX, zinc-protoporphyrin IX, and mesoporphyrin IX. Antimicrob Agents Chemother 61:e00053-17. 10.1128/AAC.00053-17. 28348154 PMC5444121

[49] Gu C, Wu Y, Guo H, Zhu Y, Xu W, Wang Y, Zhou Y, Sun Z, Cai X, Li Y, Liu J, Huang Z, Yuan Z, Zhang R, Deng Q, Qu D, Xie Y. 2020. Protoporphyrin IX and verteporfin potently inhibit SARS-CoV-2 infection in vitro and in a mouse model expressing human ACE2. Sci Bull 10.1016/j.scib.2020.12.005. PMC7724564 33318880

[50] Lu S, Pan X, Chen D, Xie X, Wu Y, Shang W, Jiang X, Sun Y, Fan S, He J. 2021. Broad-spectrum antivirals of protoporphyrins inhibit the entry of highly pathogenic emerging viruses. Bioorg Chem 107:104619. 10.1016/j.bioorg.2020.104619. 33450541 PMC7784547

[51] Lim D-S, Ko S-H, Kim S-J, Park Y-J, Park J-H, Lee W-Y. 2002. Photoinactivation of vesicular stomatitis virus by a photodynamic agent, chlorophyll derivatives from silkworm excreta. J Photochem Photobiol B 67:149–156. 10.1016/s1011-1344(02)00318-4. 12167313

[52] Almeida A, Faustino MAF, Neves M. 2020. Antimicrobial photodynamic therapy in the control of COVID-19. Antibiotics 9:320. 10.3390/antibiotics9060320. PMC7344747 32545171

[53] Sabino CP, Ball AR, Baptista MS, Dai T, Hamblin MR, Ribeiro MS, Santos AL, Sellera FP, Tegos GP, Wainwright M. 2020. Light-based technologies for management of COVID-19 pandemic crisis. J Photochem Photobiol B 212:111999. 10.1016/j.jphotobiol.2020.111999. 32855026 PMC7435279

[54] Agostinis P, Berg K, Cengel KA, Foster TH, Girotti AW, Gollnick SO, Hahn SM, Hamblin MR, Juzeniene A, Kessel D, Korbelik M, Moan J, Mroz P, Nowis D, Piette J, Wilson BC, Golab J. 2011. Photodynamic therapy of cancer: an update. CA Cancer J Clin 61:250–281. 10.3322/caac.20114. 21617154 PMC3209659

[55] de Vries RD, Schmitz KS, Bovier FT, Predella C, Khao J, Noack D, Haagmans BL, Herfst S, Stearns KN, Drew-Bear J, Biswas S, Rockx B, McGill G, Dorrello NV, Gellman SH, Alabi CA, de Swart RL, Moscona A, Porotto M. 2021. Intranasal fusion inhibitory lipopeptide prevents direct-contact SARS-CoV-2 transmission in ferrets. Science 371:1379–1382. 10.1126/science.abf4896. 33597220 PMC8011693

[56] Dubuisson J, Hsu HH, Cheung RC, Greenberg HB, Russell DG, Rice CM. 1994. Formation and intracellular localization of hepatitis C virus envelope glycoprotein complexes expressed by recombinant vaccinia and Sindbis viruses. J Virol 68:6147–6160. 10.1128/JVI.68.10.6147-6160.1994. 8083956 PMC237034

[57] van den van den Worm SHE, Eriksson KK, Zevenhoven JC, Weber F, Züst R, Kuri T, Dijkman R, Chang G, Siddell SG, Snijder EJ, Thiel V, Davidson AD. 2012. Reverse genetics of SARS-related coronavirus using vaccinia virus-based recombination. PLoS One 7:e32857. 10.1371/journal.pone.0032857. 22412934 PMC3296753

[58] Goueslain L, Alsaleh K, Horellou P, Roingeard P, Descamps V, Duverlie G, Ciczora Y, Wychowski C, Dubuisson J, Rouillé Y. 2010. Identification of GBF1 as a cellular factor required for hepatitis C virus RNA replication. J Virol 84:773–787. 10.1128/JVI.01190-09. 19906930 PMC2798365

[59] Duvet S, Chirat F, Mir A-M, Verbert A, Dubuisson J, Cacan R. 2000. Reciprocal relationship between α1,2 mannosidase processing and reglucosylation in the rough endoplasmic reticulum of Man-P-Dol deficient cells. Eur J Biochem 267:1146–1152. 10.1046/j.1432-1327.2000.01111.x. 10672024

[60] Rouillé Y, Helle F, Delgrange D, Roingeard P, Voisset C, Blanchard E, Belouzard S, McKeating J, Patel AH, Maertens G, Wakita T, Wychowski C, Dubuisson J. 2006. Subcellular localization of hepatitis C virus structural proteins in a cell culture system that efficiently replicates the virus. J Virol 80:2832–2841. 10.1128/JVI.80.6.2832-2841.2006. 16501092 PMC1395453

[61] Op De Beeck A, Voisset C, Bartosch B, Ciczora Y, Cocquerel L, Keck Z, Foung S, Cosset F-L, Dubuisson J. 2004. Characterization of functional hepatitis C virus envelope glycoproteins. J Virol 78:2994–3002. 10.1128/jvi.78.6.2994-3002.2004. 14990718 PMC353750

