# Supplementary material for: A Photoactivable Natural Product with Broad Antiviral Activity against Enveloped Viruses, Including Highly Pathogenic Coronaviruses
Source: Antimicrob Agents Chemother. 2022 Feb 15;66(2):e01581-21. doi: 10.1128/AAC.01581-21 (PMC8846325; doi:10.1128/AAC.01581-21)
Supplement: Supplemental file 1 — Supplemental material. Download AAC.01581-21-s0001.pdf, PDF file, 0.2 MB [file aac.01581-21-s0001.pdf]

SUPPLEMENTARY DATAS

Materials and methods

Chemicals

Camostat and E64D were purchased from Sigma (St. Louis, MO).

Bioguided fractionation of Mo extract

Mallotus oppositifolius was extracted with three solvents of increasing polarity. For each solvent, 5 mL/g of dried powder was used for 24h. Each extraction was repeated 3 times and the resulting extracts were combined and vacuum dried. First, the three dried leave was macerated in methylene chloride (MC), then the first ground was macerated in methanol, and finally ethanol/water (50:50) was used to extract the second ground. These 3 dried extracts were dissolved in DMSO and tested against HCoV-229E-Luc. The MC partition (most active) was fractionated by Centrifugal Partition Chromatography (CPC, Armen instruments®). CPC is a liquid/liquid chromatography based on the partition of a sample in a biphasic immiscible liquid system. The system consists of a rotor connected to two Shimadzu®-LC-20AP pumps, a CBM-20A controller, and a SPD-M20A diode array detector. The Arizona S system (heptane/ethyl acetate/methanol/water, 5:2:5:2) composed the mobile and stationary phases. Injections were carried out with 3 g of the MC extract solubilized in 50 mL of a mixture of mobile and stationary phases (5:5). The analysis took 60 min at 30 mL/min and 1200 rpm. The extrusion took 35 min at 50 mL/min with the same rotor speed. This method allowed to obtain 10 different fractions (F1-10) that were vacuum dried, dissolved in DMSO and then tested again on HCoV-229E-Luc.

Fraction F7 was the most active and selected for further fractionation by another chromatography. We used a preparative HPLC system composed of the same pumps, controller and detector as our CPC system. The stationary phase was a Vision HT HL C18 (5µm, 250×10 mm) column (Grace). The mobile phase was a mixture of methanol and water with the following gradient: 50-100% (0–15 min), and 100% methanol (15-30 min). F7 was dissolved in methanol and injected repeatedly (500 µL at 20 mg/mL). The flow rate was set at 3 mL/min. This process led to 9 partitions (7.1-7.9).

Structural elucidation of compound in F7.7

Partition 7.7 was the most active against HCoV-229E and further analysed by Ultra-High Performance Liquid Chromatography (UPLC-UV-MS) and Nuclear Magnetic Resonance (NMR). UPLC-UV-MS analysis were performed on an Acquity UPLC®H-Class system (Waters, Guyancourt, France) coupled with a Diode Array Detector (DAD) and a QDa ESI-Quadrupole Mass Separation, using an ACQUITY UPLC® BEH C18 1.7µm (2.1x100mm) column (Waters, Milford MA). Gradient elution was performed with (A) 0.1% formic acid in water and (B) 0.1% formic acid in acetonitrile at a flowrate of 0.3 mL/min, as following: 30-90% (0-3 min), 90-100% (3-7 min) before returning to the initial conditions (30% B). Analytes were monitored using UV detection (190 to 790 nm) and MS-Scan from 100 to 1000 Da (both in positive and negative mode). All data were acquired and processed using Empower 3 software.

The structural elucidation of F7.7 was conducted with NMR. Monodimensional spectra (1H and 13C) were recorded on a Bruker DPX-500 spectrometer. The chemical structure was established by comparison with literature data (1).

Cell toxicity assay

6×10^4 Huh-7, Vero-E6 and Vero-81 cells were seeded in 96-well plates and incubated for 16 h at 37˚C 5% CO_2 incubator. The cells were then treated with increasing concentrations of the compound of interest. One hour after inoculation, cells were either left in the incubator (dark condition) or taken out to be exposed to the white light of the biosafety cabinet (BSC) for 10 min (light condition), after which cells were further incubated in the dark at 37˚C 5% CO_2 for 23 h. BSC's light source lamp consists of one fluorescent tube of 36W, 3350 lumen white light. An MTS [3-(4,5-dimethylthiazol-2-yl)-5-(3-carboxymethoxyphenyl)-2-(4-sulfophenyl)-2H-tetrazolium]-based viability assay (Cell Titer 96 Aqueous non-radioactive cell proliferation assay, Promega) was performed as recommended by the manufacturer. The absorbance of formazan at 490 nm was detected using a plate reader (ELX 808 Bio-Tek Instruments Inc). Each measure was performed in triplicate and each experiment was repeated at least 3 times.

Reference

1. Cheng HH, Wang HK, Ito J, Bastow KF, Tachibana Y, Nakanishi Y, Xu Z, Luo TY, Lee KH. 2001. Cytotoxic pheophorbide-related compounds from Clerodendrum calamitosum and C. cyrtophyllum. J Nat Prod 64:915–919.

Figure S1

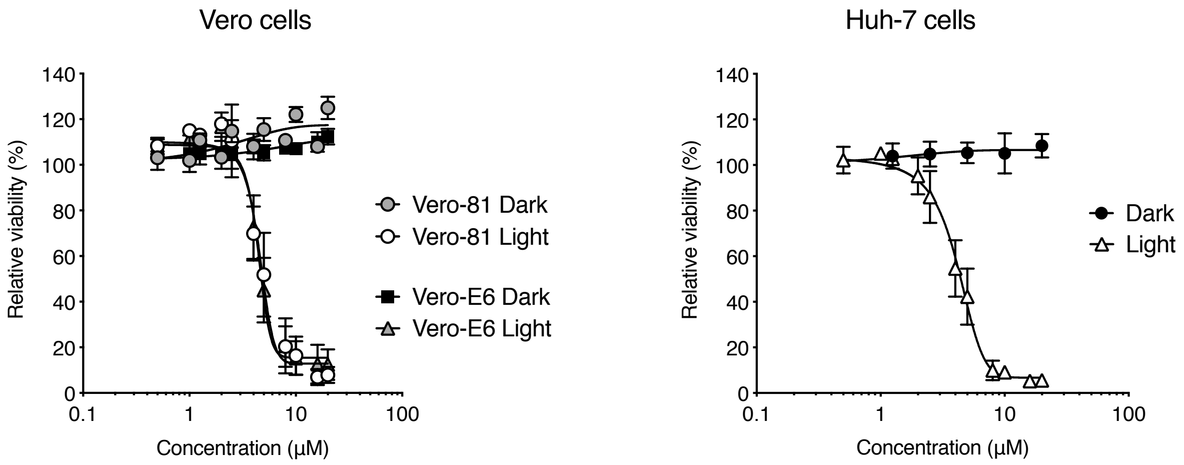

Figure S1. Toxicity of Pba depends on light exposure. Cells were incubated with Pba at different concentrations and either kept in the incubator for 24 h (Dark), or were taken out of the incubator after 1 h of incubation with Pba, and left for 10 min under light exposure, after which the cells were replaced in the incubator for 23 h (Light). Data are expressed relative to the control DMSO. Results are expressed as mean ± SEM of 3 experiments.

Figure S2

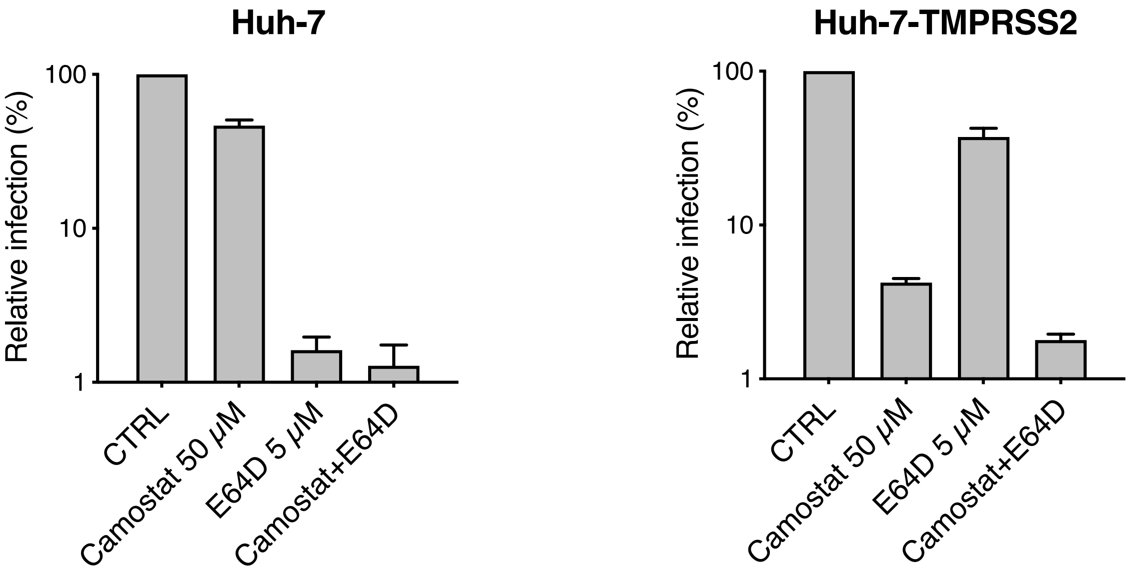

Figure S2. HCoV-229E entry pathways are inhibited by specific inhibitors. Huh-7 and Huh-7-TMPRSS2 cells were inoculated with HCoV-229E-Luc in the presence or absence of Camostat 50 µM or E64D at 5 µM or a combination of both for 1h. Inoculum and inhibitors were removed and replaced with culture medium for 6h, the cells were lyzed and luciferase activity quantified. Data are expressed relative to the control DMSO. Results are expressed as mean ± SEM of 3 experiments.

Figure S3

A

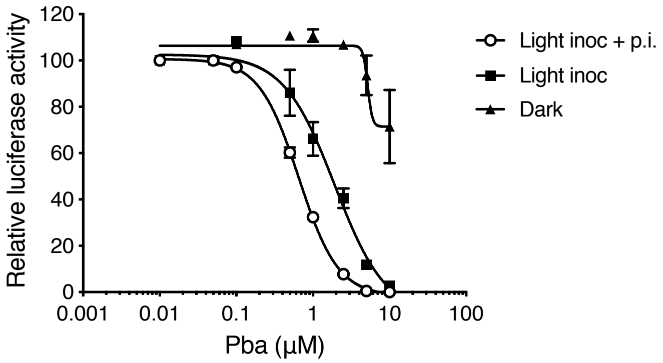

Figure S3. The antiviral activity of Pba is mainly light-dependent. Huh-7 cells were inoculated with HCoV-229E-Luc in the presence of various concentrations of Pba either with the light of the laminar flow cabinet turned on (Light inoc) or off (Dark). One hour after inoculation, the inoculum was removed, either in light (Light inoc + p.i.) or dark conditions (Light inoc), and cells were further incubated with Pba for 6 h, after which luciferase activity was measured.

Figure S4

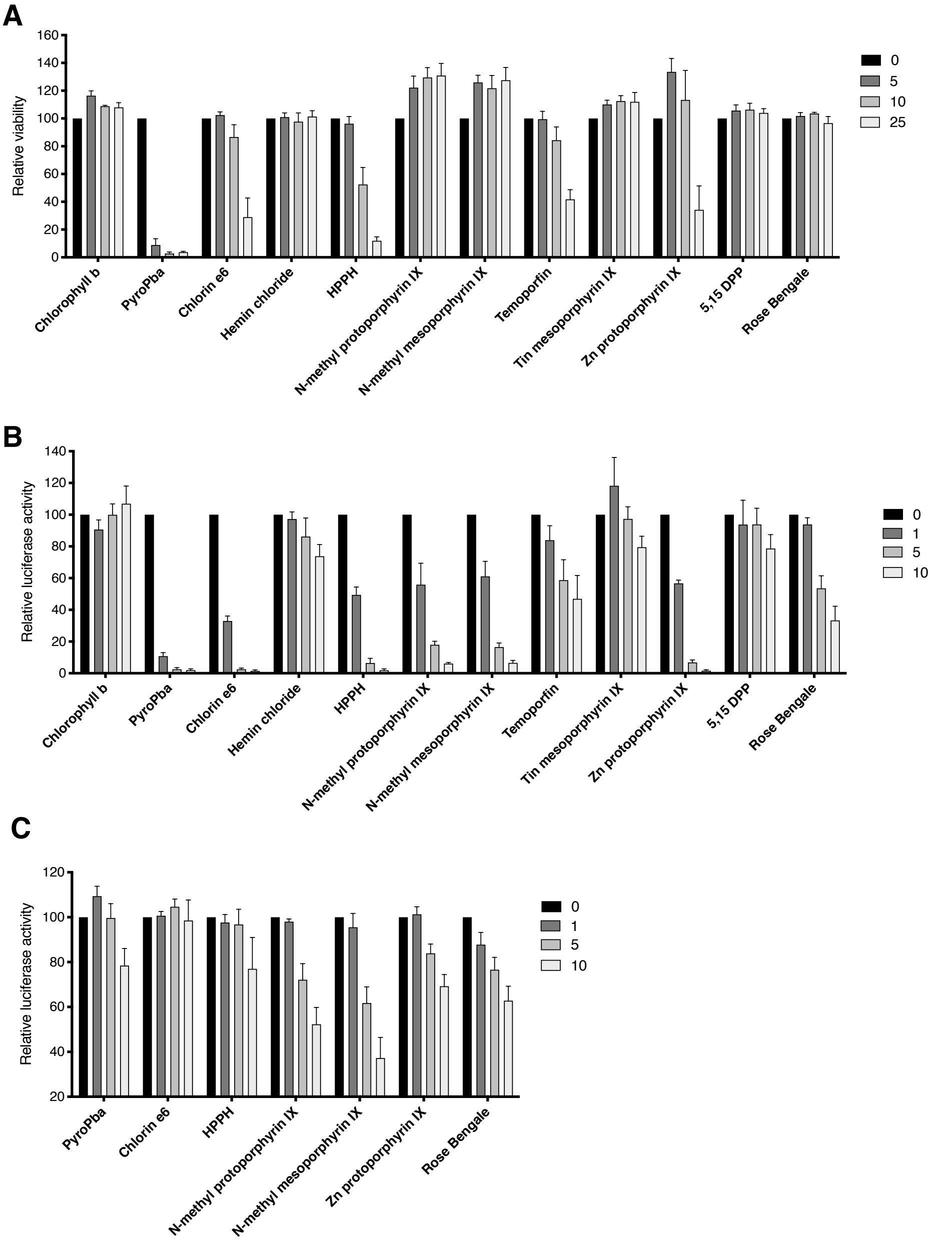

Figure S4. Activity of structurally-related Pba compounds and other photosensitizers on HCoV-229E infection. A. The toxicity on Huh-7 cells of the different compounds was determined by MTS assay. Huh-7 cells were incubated with the molecules at 5, 10, and 25 µM under the light of the cabinet. The medium was removed after 1 h and exposed for 10 min to the light of the cabinet to mimic infection assay, then placed in the dark for 23 h and MTS assay was performed. **B** and **C**. Huh-7 cells were inoculated with HCoV-229E-Luc in the presence of indicated compounds at different concentrations either under light exposure (**B**) or in the dark (**C**). 1 h post inoculation, the inoculum was removed and replace with fresh medium containing the compounds and exposed or not for 10 min to the light of the cabinet. Cells were lysed 7 h p.i. to quantify luciferase. Data are expressed relative to the control DMSO. Results are expressed as mean ± SEM of 3 experiments.
